# Global, regional, and national burdens of leukemia from 1990 to 2019: A systematic analysis of the global burden of disease in 2019 based on the APC model

**DOI:** 10.1002/cam4.7150

**Published:** 2024-09-09

**Authors:** Xiang Qu, Anjie Zheng, Jie Yang, Jinru Zhang, Hongmei Qiao, Fan Jiang, Jie Zhao, Chunping Wang, Peng Ning

**Affiliations:** ^1^ Xi'an Daxing Hospital Xi'an China; ^2^ Department of Oncology Baoji Gaoxin Hospital Baoji China; ^3^ School of Public Health Shandong Second Medical University Weifang China

**Keywords:** age‐period‐cohort model, burden of disease, disability‐adjusted life years, estimated annual percentage change, leukemia

## Abstract

**Background:**

Leukemia is the tenth most common cause of cancer death worldwide and one of the most important causes of disability. To understand the current status and changing trends of the disease burden of leukemia at the global, regional, and national levels, and to provide a scientific basis for the development of leukemia prevention and treatment strategies.

**Methods:**

Based on open data from the Global Burden of Disease Study 2019 (GBD 2019), R software was used to calculate estimated annual percentage changes to estimate trends in the age‐standardized incidence (ASIR) and the age‐standardized disability‐adjusted life years (DALY) rate due to leukemia and its major subtypes from 1990 to 2019.

**Results:**

In 2019, globally, the number of incidences and DALYs of leukemia were 643.6 × 10^3^ (587.0 × 10^3^, 699.7 × 10^3^) and 11,657.5 × 10^3^ (10529.1 × 10^3^, 12700.7 × 10^3^), respectively. The ASIR (estimated annual percentage change (EAPC) = −0.37, 95%UI −0.46 to −0.28) and the age‐standardized DALY rate (EAPC = −1.72, 95%UI −1.80 to −1.65) of leukemia showed a decreasing trend from 1990 to 2019. The APC model analysis showed that the age effect of leukemia risk was a “U”‐shaped distribution of relative risk (RR) with increasing age from 1990 to 2019, globally. The time effect was an increase in incidence rate with increasing years but a decrease in DALY rate with increasing years. The cohort effects of both incidence and DALY rates tended to increase and then decrease with the development of the birth cohort. In 1990 and 2019, smoking, high body‐mass index, occupational exposure to benzene, and occupational exposure to formaldehyde were risk factors for DALY in leukemia, especially in areas with high SDI.

**Conclusions:**

From 1990 to 2019, the disease burden of leukemia showed a decreasing trend, but it is worth noting that its overall severity is still very high. The disease burden of leukemia varies greatly from region to region, and exclusive strategies for the prevention and treatment of leukemia should be developed according to the economic and cultural development of each region.

## 1 INTRODUCTION

Leukemia is the tenth most common cause of cancer death worldwide and one of the most important causes of disability.^1^ Leukemia is a kind of malignant clonal disease caused by the malignant transformation of hematopoietic stem and progenitor cells in the hematopoietic system, and a large number of leukemia cells proliferate and accumulate in the bone marrow and other hematopoietic tissues, infiltrating organ tissues, so that normal hematopoiesis is inhibited.^2^, ^3^ Acute lymphoid leukemia (ALL), acute myeloid leukemia (AML), chronic lymphocytic leukemia (CLL), and chronic myeloid leukemia (CML) are the main subtypes of leukemia. In recent years, with the development of prevention, early diagnosis, new treatment strategies, and targeted drugs, the morbidity and mortality of leukemia have decreased, and the quality of life has been significantly improved.^4^, ^5^, ^6^, ^7^ However, leukemia is still a highly epidemic disease, which not only causes a heavy personal burden but also increases the economic cost and affects the economic structure of families and countries.^8^ It has become a serious public health problem worldwide. Leukemia, which accounts for approximately 3.4% of all new cancer cases and 3.8% of all cancer deaths in 2020, according to the Surveillance,^8^, ^9^ Epidemiology, and End Results Plan (SEER), has become a key target of the third Sustainable Development Goal (SDG), which plans to reduce premature mortality from non‐communicable diseases by one‐third by 2030.^10^

The results of the Global Burden of Disease Study 2019 (GBD 2019) showed that in 2019, there were 1,000,823 people suffering from leukemia in the world, with 60,380 deaths and 2.309 million disability‐adjusted life years (DALY) caused by leukemia.^11^, ^12^, ^13^ Clarifying the disease burden of leukemia at global, regional, and national levels in different periods will help to formulate more targeted health strategies and reduce disease risks. The study of comprehensive evaluation of the current situation and changing trends of the disease burden of leukemia at the global, regional, and national levels is essential for the formulation of national public policies, especially in countries with high or increased morbidity. However, although previous studies have systematically analyzed the disease burden of leukemia from 1990 to 2019, their study scope was limited. Evaluating data collected from other available studies can provide valuable decision support to policymakers, identify effective disease control strategies to minimize disease burden, and help researchers fill knowledge gaps.

Our study proposes to further assess the disease burden of leukemia based on GBD 2019 data by identifying temporal trends in incidence and DALY rates of leukemia from 1990 to 2019 at global, regional, and national levels. We utilized the EAPC and APC models to explore the risks of leukemia incidence and disease burden over the past 30 years at the global, regional, and national levels. In addition, we also focused on the main driving factors affecting leukemia, including smoking, high body‐mass index, occupational exposure to benzene, and occupational exposure to formaldehyde. Our findings can be used as an important extension and supplement to the previous disease burden of leukemia and help to develop leukemia prevention targeting strategies appropriate for different countries.

## 2 METHODS

### 2.1 Data source

The GBD study is a statistical report on the health status of the populations in all countries and regions of the world, integrating health indicators such as the births and deaths (including the causes of death) and the relationship between incidence and risk factors.^14^ It employs a uniform and standardized methodology to provide a scientific and comprehensive assessment of disease, injury, and risk factor across age and gender populations globally, ensuring results are comparable and nationally and regionally representative,^15^ and GBD 2019 includes estimates for 369 diseases and injuries, and 87 risk factors from 1990 to 2019, covering 204 countries.^11^, ^12^, ^13^, ^16^ This study collected the number of leukemia cases, deaths, DALYs, and corresponding age‐standardized rate by gender, region, country, and type from 1990 to 2019. All data obtained in the present study were publicly available on the Institute for Health Metrics and Evaluation (IHME) website and can be accessed with open online tools (https://ghdx.healthdata.org/). Our research is based on the analysis of age‐standardized rate to quantify the health costs of leukemia and to help policymakers assess the burden of leukemia, measure the development of specific treatments, allocate resources, and develop policies aimed at improving health systems and reducing the disease burden of leukemia over time.^8^ Ethical approval and consent to participate did not apply to this study.

### 2.2 Definition

#### 2.2.1 Leukemia

In GBD, leukemia is defined as 5 months for the diagnosis and initial treatment phase, 43.67 months for the metastatic phase, and 1 month for the advanced phase. All phases introduce their respective disability weights. Patients who have survived for more than a decade are considered cured.^17^ Leukemia is divided into four subtypes: ALL, AML, CLL, and CML. Leukemia is classified as diagnosis codes C91‐C96 by the International Statistical Classification of Diseases (10th Revision) (ICD‐10).^18^

#### 2.2.2 YLD

Year lived with disability (YLD) represents healthy life years lost among survivors and were estimated by multiplying the prevalence rate by the disability weight, YLD = Prev × DW.^19^

#### 2.2.3 YLL

Years of life lost (YLL) were estimated by the number of age‐specific leukemia deaths multiplied by the healthy life expectancy, YLL = N × L.^20^

#### 2.2.4 DALY

Disability‐adjusted life years (DALY) are the sum of years of life lost (YLL) and years lived with disability (YLD) and are calculated by the following formula: DALY = (N × L) + (Prev × DW).^21^, ^22^

#### 2.2.5 Uncertainty interval

For incidence and DALY, the corresponding 95% uncertainty intervals (UI) were estimated using the 2.5th and 97.5th estimates in posterior simulation of 1000 ordered draws, with the aim of testing the uncertainty distribution derived from random and systematic errors. Significant differences were defined as no overlap between any two estimates of the 95% UI.^23^, ^24^

### 2.3 Statistical analysis

In order to determine time trends across regions and countries, the age standardized rate (per 100,000 people) was calculated using the global age‐standard population established by the World Health Organization. We used R 4.3.2 software to conduct EAPC analysis, using time as the independent variable and the age‐standardized rate of incidence and DALY as the dependent variable, respectively, to quantify the trends in leukemia incidence and DALY. Using Stata 16 software, the age‐period‐cohort (APC) model based on its intrinsic estimation algorithm was used to estimate the age effect, period effect, and cohort effect of leukemia incidence and DALY. The test level is *α* = 0.05.

#### 2.3.1 ASR

Age‐standardized rates (ASR) are necessary when comparing the age distribution of several populations with different age structures and the same population over time. The ASR (per 100,000 population) is obtained by adding the product of the age‐specific rate ($a_{i}$, *i* indicates the age category) and the number of people (or weights) ($\;W_{i}$) in the same age subgroup i of the selected reference standard population and then dividing by the sum of the standard population weights.^25^ ASR and 95%UI were calculated based on the GBD2019 global age standard population. Trends in ASR can better proxy for shifts in disease patterns and changing risk factors in a population, as well as assess the effectiveness of current prevention strategies and recommend more targeted strategies when necessary.^26^

$$\mathrm{ASR}=\frac{\sum_{i=1}^{A}a_{i}W_{i}}{\sum_{i=1}^{A}W_{i}}\times 100,000$$

#### 2.3.2 EAPC

An indicator of EAPC is used to reflect the temporal trend of the ASR.^27^ EAPC is a summary and widely used indicator of rate trends over a specified interval, which can be calculated from a regression model fitted to the natural logarithm of the rate, That is, y=a+bx+e, where $y=\ln\left(\mathrm{ASR}\right)$, x = year, EAPC was defined as $\text{EAPC}=100\times \left(\exp\left(\beta \right)-1\right).$ ^28^ The 95% UI of EAPC was also obtained from the linear regression model.^29^ When both the lower limit of EAPC and its 95% UI are greater than 0, ASR is considered to be on an increasing trend, and conversely, when both the estimated value of EAPC and its 95% UI upper limit are less than 0, ASR is on a decreasing trend; otherwise, ASR is considered to be stable over time.

#### 2.3.3 Age‐period‐cohort model

The age‐period‐cohort model (APC) is widely used to estimate the effect of three independent factors of age, period and cohort on disease incidence or mortality.^30^ It is based on the Poisson distribution and improves the traditional descriptive analysis method by decomposing the target analysis variables into three dimensions: age, period, and cohort, so as to analyze the long‐term trend of disease changes over time.^31^, ^32^ The age effects refer to differences in disease incidence and DALY rates among different age groups; the period effects refer to changes in disease incidence and DALY rates in populations affected by human factors such as the development of disease diagnostic techniques, screening, and early detection; and the cohort effects refer to changes in disease incidence and DALY rates due to different risk factors to which people are exposed in different generations.^33^ The basic expression is: $\text{In incidence}=\mu +\alpha_{a}+\beta_{b}+\gamma_{c}+\varepsilon_{\mathrm{abc}}$, where In incidence denotes the natural logarithm of global incidence of leukemia, μ is the intercept term, $\alpha_{a}$ is the age effect for the ath age group, $\beta_{b}$ is the period effect for the bth period group, $\gamma_{c}$ is the cohort effect for the c birth cohort, and $\varepsilon_{\mathrm{abc}}$ denotes the error term or residual term.^34^ Since age, period, and cohort have a perfectly linear relationship, there is the problem of model failure to identify them. In this study, the intrinsic estimator (IE) algorithm is used to solve this multicollinearity problem,^35^, ^36^ which does not require the researcher to make the model assumptions in advance and has the characteristics of estimability and unbiasedness.^37^

### 2.4 Visual analysis of data

All data visualization is done by ArcGIS (Environmental Systems Research Institute, Inc., USA), R 4.2.3 (R Foundation for Statistical Computing), STATA 16.0 (Stata Corp., College Station, TX, USA), and Excel 2003. *p* < 0.05 was considered statistically significant in our study.

### 2.5 Geographical estimation

#### 2.5.1 Socio‐demographic index

The socio‐demographic index (SDI) is a composite indicator of a country's lagged distribution of per capita income, average years of education, and female fertility under the age of 25.^11^ The SDI is a summary indicator derived from measures of per capita income, educational attainment, and fertility.^38^ In a nutshell, SDI is a weighting of each component that is rescaled between 0 and 1. SDI = 1.0 can be interpreted as the regions with the highest observed educational attainment, the highest logarithm of per capita income, and the lowest fertility rate. Countries were divided into five groups based on the SDI index: high SDI regions (>0.81), high–middle SDI regions (0.70–0.81), middle SDI regions (0.61–0.69), low–middle SDI regions (0.46–0.60), and low SDI regions (<0.46). The formula for SDI: $I_{\mathrm{Cly}}=\left(C_{\mathrm{ly}}-C_{\mathrm{low}}\right)/\left(C_{\text{high}}-C_{\mathrm{low}}\right)$, where $I_{\mathrm{Cly}}$ is the exponential value of the covariates C, position l, and year y. The method of SDI generation has been described in detail in the previous literature.^39^, ^40^ The SDI index for 1990–2019 is available from the GBD website (http://ghdx.healthdata.org/record/ihme‐data/gbd‐2019‐socio‐demographic‐index‐sdi‐1950‐2019).

## 3 RESULTS

### 3.1 The incidence rate of leukemia and its changing trends

Globally, the number of cases of leukemia increased from 474.9 × 10^3^ (95%UI 388.6 × 10^3^, 560.6 × 10^3^) to 643.6 × 10^3^ (95%UI 587.0 × 10^3^, 699.7 × 10^3^) from 1990 to 2019, with an increase rate of 35.5%; however, the ASIR showed a decreasing trend from 9.6/100,000 in 1990 to 8.2/100,000 in 2019, with a decrease rate of 14.58% (EAPC = −0.37, 95%UI −0.46 to −0.28). Compared to Female, man were more likely to have leukemia (man‐to‐female ratio 1.14:1 in 1990, 1.31:1 in 2019), and the downward trend was lower in man. (Man EAPC = −0.06, 95%UI −0.12 to 0.00, Female EAPC = −0.71, 95%UI −0.83 to −0.59; Table 1).

**TABLE 1:** Incidence of leukemia and its trends in 1990 and 2019.

Characteristics	1990		2019		1990–2019
	Incidence cases (95% UI)	ASR per 100,000 No. (95% UI)	Incidence cases (95% UI)	ASR per 100,000 No. (95% UI)	EAPC No. (95% CI)
Global	474923.8 (388558.6560550.3)	9.6 (8.1,11.0)	643579.0 (586980.1699729.4)	8.2 (7.5,8.9)	−0.37 (−0.46, −0.28)
Sex					
Male	241301.2 (184752.2294464.2)	10.4 (8.4,12.2)	350582.3 (307569.5389659.9)	9.4 (8.3,10.5)	−0.06 (−0.12,0.00)
Female	233622.6 (184621.6289890.6)	9.1 (7.4,11.0)	292996.7 (263380.3322329.8)	7.2 (6.5,8.0)	−0.71 (−0.83, −0.59)
Sociodemographic index					
High SDI	104370.9 (100125.2106822.3)	11.2 (10.7,11.5)	188538.0 (169161.6208210.8)	12.0 (10.9,13.2)	1.13 (1.00, 1.27)
High–middle SDI	114946.6 (99419.9127235.0)	10.5 (9.1,11.7)	167724.9 (150621.9183328.6)	10.1 (9.1,11.1)	0.41 (0.30, 0.53)
Middle SDI	149075.1 (113,016,179036.5)	9.0 (7.2,10.6)	163718.7 (144033.1183739.1)	6.9 (6.1,7.7)	−1.05 (−1.25, −0.84)
Low–middle SDI	69811.1 (46706.497160.7)	6.3 (4.6,8.1)	74170.7 (64985.186007.1)	4.7 (4.2,5.5)	−1.50 (−1.61, −1.39)
Low SDI	36470.3 (20049.960212.1)	6.4 (4.4,9.4)	49048.9 (37936.660801.8)	5.1 (4.1,6.0)	−1.49 (−1.56, −1.42)
Region					
Andean Latin America	2720.0 (2215.33528.2)	7.4 (6.3,9.4)	4423.5 (3233.65622.2)	7.3 (5.3,9.3)	−0.03 (−0.18, 0.11)
Australasia	2179.6 (2074.82277.5)	9.8 (9.3,10.2)	4717.2 (3814.45798.7)	10.5 (8.4,12.8)	1.17 (1.03, 1.31)
Caribbean	2771.6 (2097.93834.7)	8.2 (6.5,10.9)	3607.1 (2835.44446.1)	7.6 (5.8,9.5)	0.05 (−0.11, 0.21)
Central Asia	6368.1 (5722.16840.1)	8.9 (8.1,9.4)	5246.7 (4605.66047.7)	6.0 (5.3,6.9)	−2.02 (−2.17, −1.88)
Central Europe	10480.5 (10183.210868.3)	7.9 (7.7,8.2)	17318.4 (15236.619601.5)	9.6 (8.4,10.9)	2.23 (2.15, 2.31)
Central Latin America	11100.2 (10544.911662.1)	7.0 (6.8,7.3)	15977.4 (13603.318616.8)	6.6 (5.6,7.7)	−0.16 (−0.22, −0.10)
Central Sub‐Saharan Africa	3303.7 (1491.0,6032.2)	5.5 (3.6,8.2)	4091.4 (3061.0,5533.0)	3.9 (2.9,5)	−2.05 (−2.17, −1.93)
East Asia	147555.6 (106054.6178262.7)	12.7 (9.2,15.2)	159358.3 (131903.5185876.7)	10.4 (8.7,12.3)	−0.73 (−1.07, −0.38)
Eastern Europe	19674.2 (18893.120412.9)	8.2 (7.8,8.5)	21615.3 (19527.923850.5)	7.5 (6.9,8.3)	0.36 (0.14, 0.58)
Eastern Sub‐Saharan Africa	19118.2 (10211.833790.0)	8.3 (5.3,13.2)	21547.9 (13995.329459.8)	5.9 (4.2,7.7)	−2.05 (−2.17, −1.93)
High‐income Asia Pacific	15845.6 (14849.416732.3)	9.0 (8.4,9.6)	29195.9 (24933.133270.9)	10.3 (9,11.7)	1.95 (1.85, 2.05)
High‐income North America	39060.8 (37173.440103.1)	11.8 (11.3,12.1)	61549.3 (53737.770238.0)	10.7 (9.4,12.1)	0.41 (0.33, 0.50)
North Africa and Middle East	29191.7 (20053.839886.2)	9.5 (7.1,12)	39297.5 (32616.645056.0)	7.8 (6.5,8.8)	−0.95 (−1.06, −0.83)
Oceania	407.9 (272.4607.5)	7.1 (5.2,9.8)	808.0 (533.0,1224.9)	6.8 (4.8,9.7)	−0.16 (−0.26, −0.06)
South Asia	46309.2 (30339.364723.6)	4.7 (3.6,6.1)	59855.9 (51906.570104.6)	3.8 (3.3,4.5)	−1.06 (−1.19, −0.93)
Southeast Asia	37667.4 (25233.953044.3)	8.6 (6.3,11.3)	42262.8 (35843.549692.3)	6.8 (5.8,8)	−0.86 (−0.93, −0.79)
Southern Latin America	3607.9 (3476.93735.1)	7.5 (7.2,7.8)	5386.8 (4272.56699.7)	7.2 (5.7,8.9)	0.27 (0.19, 0.34)
Southern Sub‐Saharan Africa	1875.2 (1607,2140.0)	4.6 (4,5.1)	2763.2 (2383.0,3135.1)	4.3 (3.7,4.9)	−0.07 (−0.13, 0.00)
Tropical Latin America	9348.1 (8706.19989.8)	6.9 (6.5,7.2)	12371.1 (11556.913082.1)	5.5 (5.1,5.9)	−0.19 (−0.28, −0.11)
Western Europe	60011.2 (57773.961717.1)	12.7 (12.3,13.1)	118623.6 (102900.9135440.5)	16.9 (14.7,19.4)	1.68 (1.40, 1.97)
Western Sub‐Saharan Africa	6326.9 (4290.19213.1)	3.8 (3,4.8)	13561.9 (10265.0,17280.8)	3.9 (3.1,4.7)	−0.19 (−0.24, −0.13)

*Note*: The unit of measurement is 100,000. The data in parentheses is 95% uncertainty interval.

Abbreviations: ASR, age standardized rate; CI, confidence interval; EAPC, estimated annual percentage change; UI, uncertainty interval; SDI, socio‐demographic index.

**FIGURE 1:**
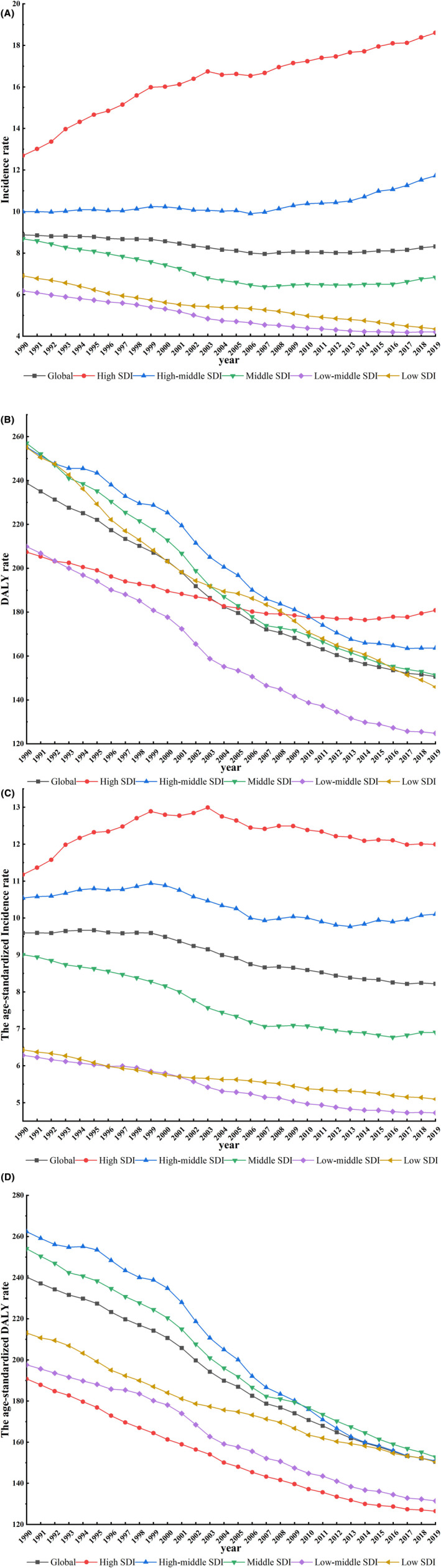
Global compared with the different SDI regions for Incidence (A), DALY rate (B), ASIR (C), and the age‐standardized DALY rate (D). The Y‐axis represents the disease burden globally and in different SDI regions, and the X‐axis represents year. Four figures depict disease burden for different indicators in different regions. ASIR, age standardized incident rate; DALY, disability‐adjusted life years; SDI, socio‐demographic index.

At the regional level, the age‐standardized incidence burden of leukemia was highest in high SDI regions, rising from 11.2 (95%UI 10.7 to 11.5) in 1990 to 12.0 (95%UI, 10.9 to 13.2) in 2019, EAPC = 1.13 (95%UI 1.00 to 1.27). The age‐standardized incidence burden of leukemia declined most rapidly in low and middle SDI regions, EAPC = −1.50 (95%UI −1.61 to −1.39; Figure 1A,C).

Subgroup analysis by geographic region showed that the number of leukemia cases was highest in East Asia (147.6 × 10^3^ in 1990, 159.4 × 10^3^ in 2019) and Western Europe (60.0 × 10^3^ in 1990, 118.7 × 10^3^ in 2019), fastest growing in Central Europe (EAPC = 2.23, 95%UI 2.15 to 2.31), and declining fastest in Central Sub‐Saharan Africa and Eastern Sub‐Saharan Africa (both EAPC = −2.05, 95%UI −2.17 to −1.93; Table 1).

**FIGURE 2:**
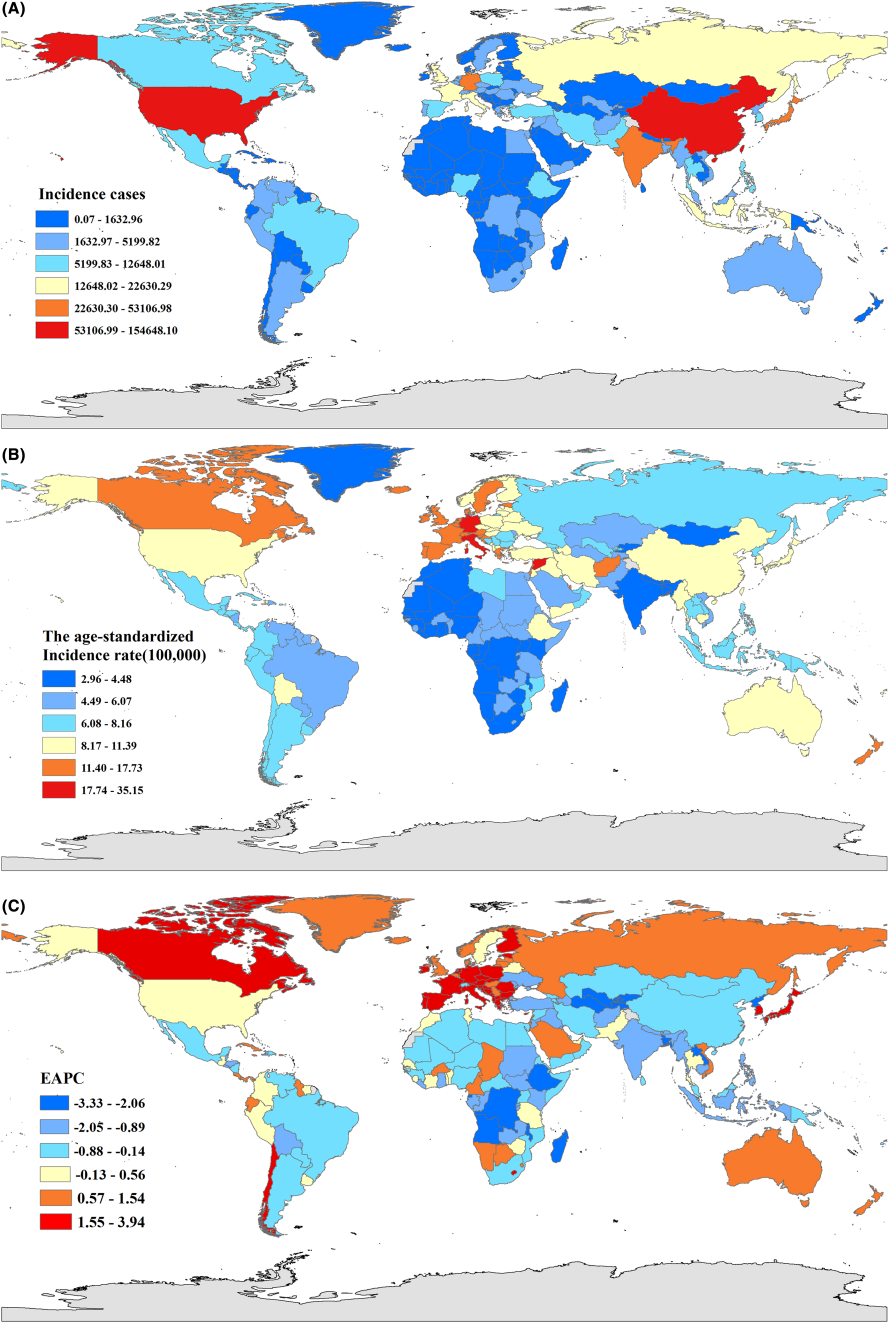
The global disease burden of Leukemia for both sexes in 204 countries and territories. (A) The number of Leukemia cases in 2019; (B) The ASIR of Leukemia in 2019; (C) The EAPC of the ASIR of Leukemia from 1990 to 2019. ASIR, age standardized incident rate; EAPC, estimated annual percentage change.

**FIGURE 3:**
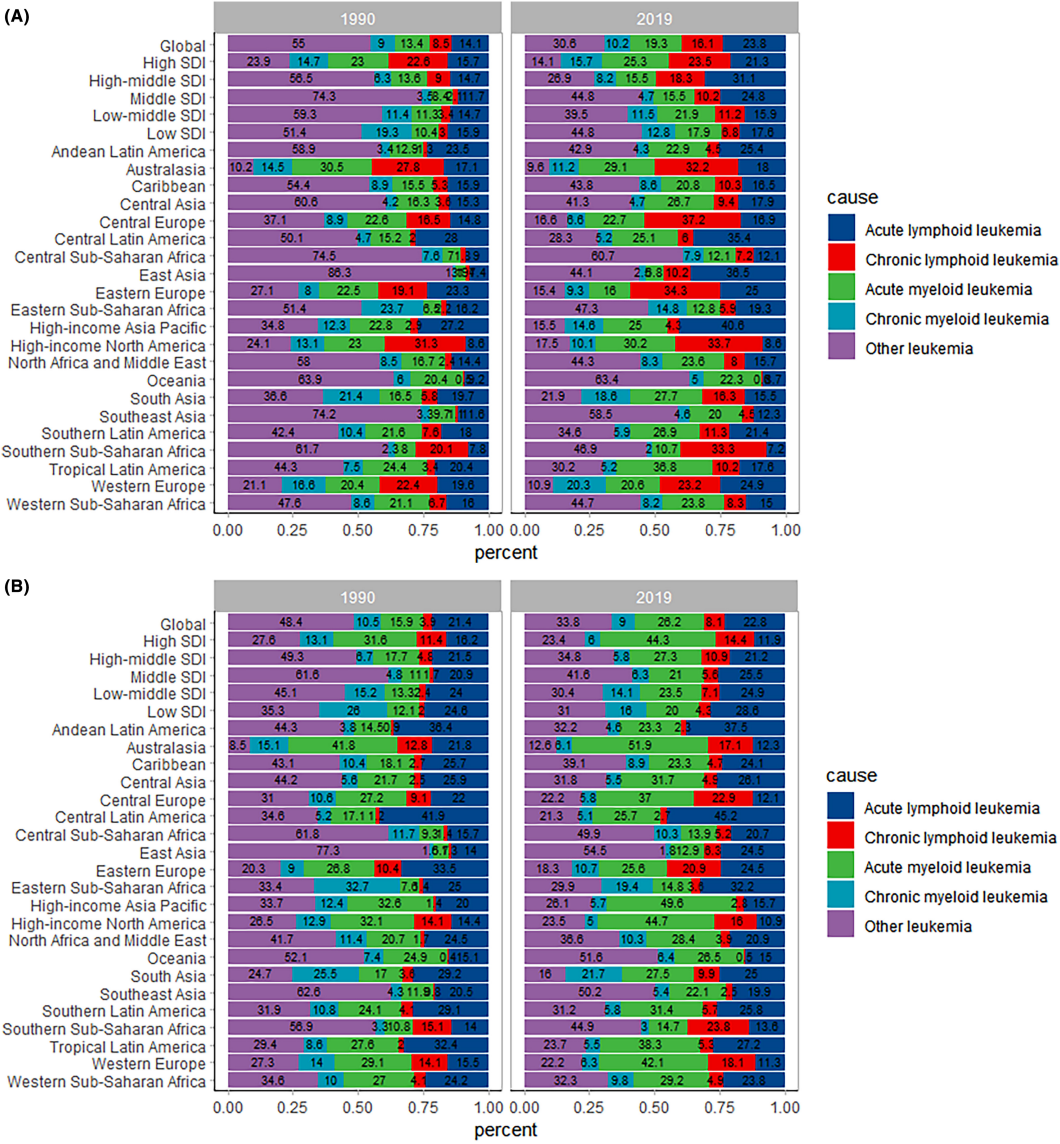
The ASIR (A) and the age‐standardized DALY rate (B) for the five subtypes of Leukemia, globally and regionally, 1990 and 2019. ASIR, Age standardized incident rate. DALY, Disability‐adjusted life years.

Among 204 countries or regions level, China, United States of America, and India had the highest number of leukemia cases (142.7 × 10^3^, 35.2 × 10^3^, and 34.0 × 10^3^ in 1990; 154.6 × 10^3^, 53.1 × 10^3^, and 43.6 × 10^3^ in 2019, respectively). The ASIR for leukemia between 1990 and 2019 were different in 204 countries and regions, with the Syrian Arab Republic (26.89, 95%UI 20.46 to 33.41), Monaco (22.51, 95%UI 17.26 to 29.14), and San Marino (22.33, 95%UI 18.01 to 28.49) having the highest ASIR in 1990; San Marino (35.14, 95%UI 26.37 to 47.19), Monaco (27.38, 95%UI 19.66 to 36.33), and Andorra (22.71, 95%UI 16.74 to 30.34) having the highest ASIR in 2019 (Figure 2). Figure 3A shows the proportions of the incidence of various leukemia subtypes at the global and regional levels in 1990 and 2019.

**FIGURE 4:**
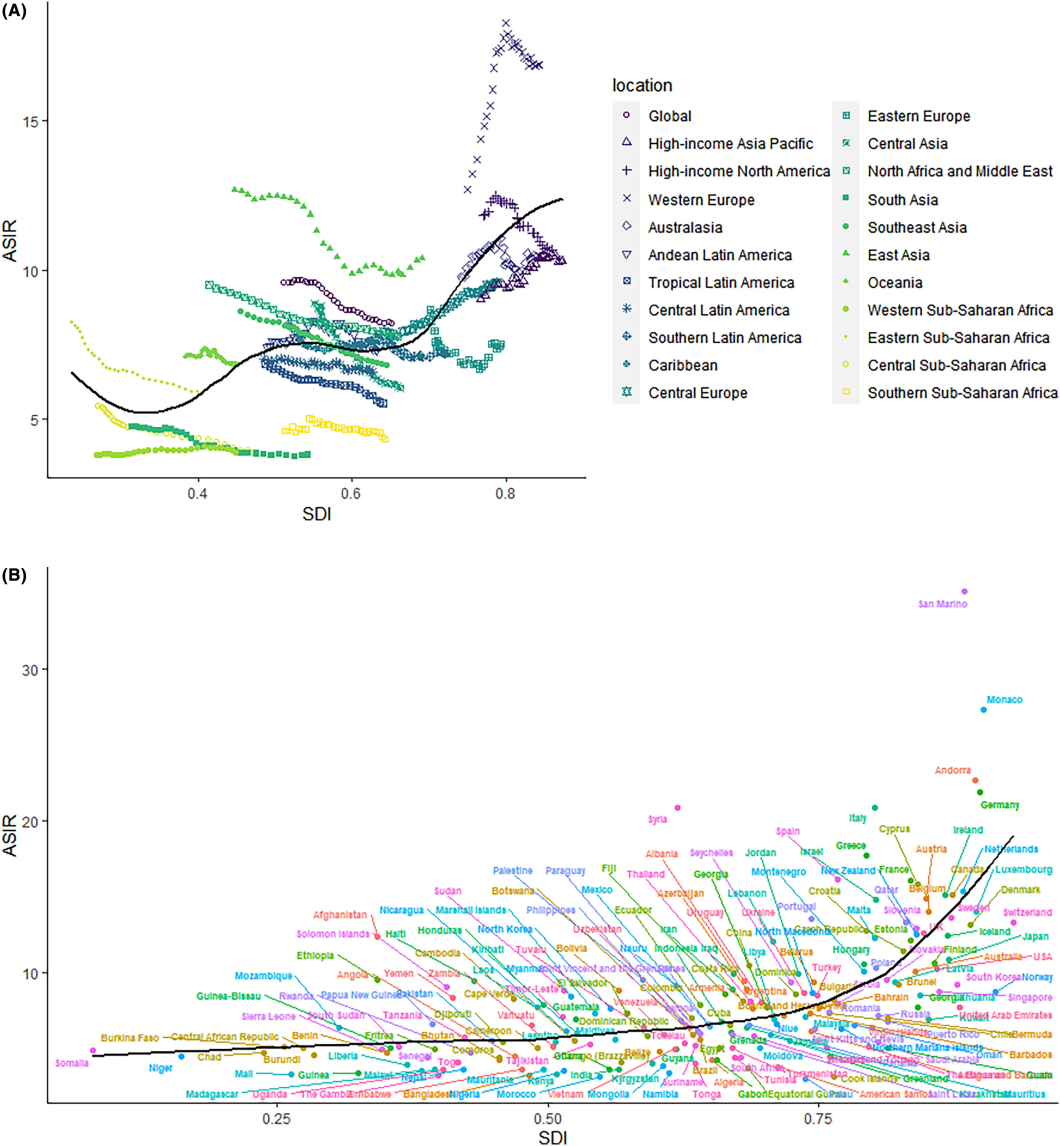
The ASIR of leukemia for 21 GBD regions (A) and 204 countries and territories (B) by Socio‐demographic Index, 1990–2019. Expected values based on Socio‐demographic Index and disease rates in all locations are shown as the black line. The black line represents the expected ASIR and SDI in 204 countries and territories. Each point shows the observed ASIR for a specified country in 2019. ASIR, Age standardized incident rate; GBD, Global Burden of Disease; SDI, Socio‐demographic index.

Figure 4 shows the observed ASIR for the regions and countries associated with the spatial data infrastructure, as well as the expected levels for each region and country based on the spatial data infrastructure. Andean Latin America, Central Latin America, Australasia, Southern Latin America, and the Caribbean followed the expected trend during the study period. In many of the middle‐SDI regions, we observed a wide variation in disease patterns, with some regions being well below expected levels with minimal changes in ASIR throughout the study period, while others were well above expected levels and showed fluctuating or decreasing trends in ASIR. In 2019, globally, with some exceptions, there was a positive correlation between ASIR and SDI for leukemia.

### 3.2 The DALY rate of leukemia and its changing trends

The number of DALYs for leukemia globally was 12,777.3 × 10^3^ (95%UI 10,707.3 × 10^3^ to 15,365.5 × 10^3^) in 1990 and 11,657.5 × 10^3^ (95%UI 10,529.1 × 10^3^ to 12,700.7 × 10^3^) in 2019, and its age‐standardized DALY rate decreased from 240.3 (95%UI 206.0 to 283.3) in 1990 to 150.5 (95%UI 135.7 to 164.1) in 2019, EAPC = −1.72 (95%UI −1.80 to −1.65). The burden of leukemia disease was higher in men than in women, and the age‐specific DALY rates showed a decreasing trend in both men and women (male: 264.3 (95%UI 198.8 to 315.4) in 1990, 175.4 (95%UI 149.6 to 196.5) in 2019; female: 219.2 (95%UI 173.1 to 263.9) in 1990, 127.2 (95%UI 115.0 to 138.9) in 2019), and the decreasing trend was higher in women (EAPC = −2.05, 95%UI −2.15 to −1.94) than in men (EAPC = −1.46, 95%UI −1.52, to −1.40; Table 2).

**TABLE 2:** DALY of Leukemia and its trends in 1990 and 2019.

Characteristics	1990		2019		1990–2019
	DALY cases (95% UI)	ASR per 100,000 No. (95% UI)	DALY cases (95% UI)	ASR per 100,000 No. (95% UI)	EAPC No. (95% CI)
Global	12777362.5 (10707316.315365541.7)	240.3 (206.0,283.3)	11657546.7 (10529142.212700651.1)	150.5 (135.7164.1)	−1.72 (−1.80, −1.65)
Sex					
Male	6912534.7 (4954492.88459811.8)	264.3 (198.8315.4)	6670586.1 (5697463.0,7460123.7)	175.4 (149.6196.5)	−1.46 (−1.52, −1.40)
Female	5864827.8 (4532435.97181122.5)	219.2 (173.1263.9)	4986960.6 (4524242.85443176.1)	127.2 (115.0,138.9)	−2.05 (−2.15, −1.94)
Sociodemographic index					
High SDI	1704284.2 (1656175.31736499.0)	190.8 (185.4194.5)	1832262.5 (1731033.91910164.4)	126.5 (120.7131.6)	−0.56 (−0.64, −0.48)
High–middle SDI	2936149.6 (2602821.63210557.8)	262.3 (233.0,286.5)	2340608.3 (2088069.92534242.1)	150.9 (133.7164.0)	−1.78 (−1.88, −1.68)
Middle SDI	4411499.4 (3686396.65127057.2)	254.0 (215.1291.7)	3627966.3 (3206480.34046086.1)	152.7 (134.8170.3)	−1.95 (−2.05, −1.85)
Low–middle SDI	2371092.3 (1666532.83222780.4)	197.6 (149.1256.2)	2201321.7 (1920873.82557600.5)	131.5 (115.0,152.6)	−1.97 (−2.06, −1.89)
Low SDI	1347328.8 (747519.62277373.5)	213.1 (140.7326.5)	1647140.9 (1308167.82021129.5)	150.4 (121.3178.6)	−1.88 (−1.94, −1.82)
Region					
Andean Latin America	94496.6 (80766.5119470.4)	237.2 (205.1295.9)	130139.3 (94644.6167107.0)	208.3 (151.9266.8)	−0.61 (−0.75, −0.47)
Australasia	38151.3 (36930.139297.7)	177.0 (171.2182.5)	49849.3 (46144.153204.2)	123.9 (116.1131.4)	−0.52 (−0.65, −0.39)
Caribbean	85896.7 (64848.2118635.7)	241.9 (189.2323.8)	93167.9 (71599.4119464.2)	200.5 (151.1261.3)	−0.60 (−0.76, −0.44)
Central Asia	191370.5 (181113.2200890.5)	262.9 (250.8274.0)	149495.5 (130533.9171440.8)	164.4 (144.5188.2)	−2.27 (−2.43, −2.12)
Central Europe	259496.7 (252999.6269688.1)	201.8 (196.0,210.4)	233129.5 (205859.9262478.3)	145.0 (127.7164.0)	−0.05 (−0.10, −0.01)
Central Latin America	381862.5 (364252.0,399434.9)	222.7 (214.5231.4)	470070.9 (403984.3543365.4)	190.6 (163.8220.6)	−0.63 (−0.67, −0.59)
Central Sub‐Saharan Africa	106878.5 (50943.2187905.6)	163.8 (106.8245.4)	132016.8 (97917.9177958.5)	110.3 (83.8139.4)	−2.08 (−2.18, −1.98)
East Asia	4001414.9 (3165129.24679038.9)	331.0 (262.6387.1)	2399037.7 (2014460.0,2777481.3)	164.1 (136.9189.2)	−2.69 (−2.84, −2.53)
Eastern Europe	549289.5 (529899.9566995.5)	241.1 (231.3249.8)	352317.0 (321016.6387489.2)	140.0 (127.8153.0)	−1.76 (−1.95, −1.56)
Eastern Sub‐Saharan Africa	719617.8 (387750.0,1321428.1)	282.0 (177.1476.4)	727912.7 (475127.7980634.8)	173.0 (121.5228.4)	−2.48 (−2.60, −2.36)
High‐income Asia Pacific	326745.4 (307456.2337291.6)	186.0 (173.5193.1)	267329.0 (241668.1284478.4)	98.2 (89.5104.9)	−0.98 (−1.12, −0.85)
High‐income North America	624591.2 (606198.4637398.8)	200.4 (194.9204.1)	738966.2 (703032.2769665.5)	143.8 (138.1149.1)	−0.48 (−0.57, −0.39)
North Africa and Middle East	901950.3 (674371.91197981.4)	270.7 (211.6338.8)	1011554.8 (822536.81173620.6)	183.4 (150.7211.2)	−1.58 (−1.65, −1.51)
Oceania	13220.8 (9539.519048.8)	210.0 (158.5288.0)	24621.7 (17036.736614.3)	192.0 (137.2272.2)	−0.37 (−0.46, −0.28)
South Asia	1768784.5 (1171335.12491868.5)	157.6 (116.1208.0)	1847835.0 (1598397.82192213.6)	109.2 (94.3129.6)	−1.85 (−1.96, −1.73)
Southeast Asia	1164684.2 (826793.91583685.6)	249.5 (187.6321.8)	1156787.7 (996181.61344034.4)	178.9 (153.9208.0)	−1.29 (−1.34, −1.25)
Southern Latin America	107639.0 (104300.8111074.6)	218.7 (212.1225.5)	117863.7 (111407.2124877.4)	166.9 (157.6177.3)	−0.79 (−0.85, −0.73)
Southern Sub‐Saharan Africa	55770.1 (49236.963506.0)	123.7 (110.2137.8)	71812.0 (62129.481633.2)	103.0 (88.1115.6)	−0.53 (−0.67, −0.40)
Tropical Latin America	304560.0 (284942.0,327332.1)	204.3 (192.9217.2)	322893.1 (303422.1342799.7)	146.7 (137.0,157.3)	−0.95 (−1.01, −0.89)
Western Europe	850380.6 (826175.6869268.0)	190.0 (185.4194.6)	893135.4 (828830.9940341.7)	130.5 (123.3137.0)	−0.31 (−0.37, −0.25)
Western Sub‐Saharan Africa	230561.3 (154372.4341036.2)	119.3 (89.6156.4)	467611.3 (348733.4610675.2)	113.0 (88.9139.8)	−0.43 (−0.48, −0.37)

*Note*: The unit of measurement is 100,000. The data in parentheses is 95% uncertainty interval.

Abbreviations: ASR, age standardized rate; CI, confidence interval; DALY, disability‐adjusted life years; EAPC, estimated annual percentage change; UI, uncertainty interval; SDI, socio‐demographic index.

Leukemia DALYs were highest in the middle SDI regions (1990: 44.1 × 10^5^, 95%UI 36.9 × 10^5^ to 51.2 × 10^5^, 2019: 36.3 × 10^5^, 95%UI 32.1 × 10^5^ to 40.5 × 10^5^), and the decreasing trend was highest in the low–middle SDI regions (EAPC = −1.97, 95%UI −2.06 to −1.89; Figure 1B,D).

Analysis of the 22 GBD geographic regions indicated that East Asia and South Asia had the highest DALYs for leukemia. (East Asia: 40.0 × 10^5^ (31.7 × 10^5^, 46.8 × 10^5^) in 1990, 24.0 × 10^5^ (20.1 × 10^5^, 27.8 × 10^5^) in 2019; South Asia: 17.7 × 10^5^ (11.7 × 10^5^, 24.9 × 10^5^) in 1990, 18.5 × 10^5^ (16.0 × 10^5^, 22.0 × 10^5^) in 2019). All geographic regions showed a decreasing trend in age‐labeled DALY rates for leukemia, with the highest decreasing trend in East Asia (EAPC = −2.69, 95%UI −2.84 to −2.53; Table 2).

**FIGURE 5:**
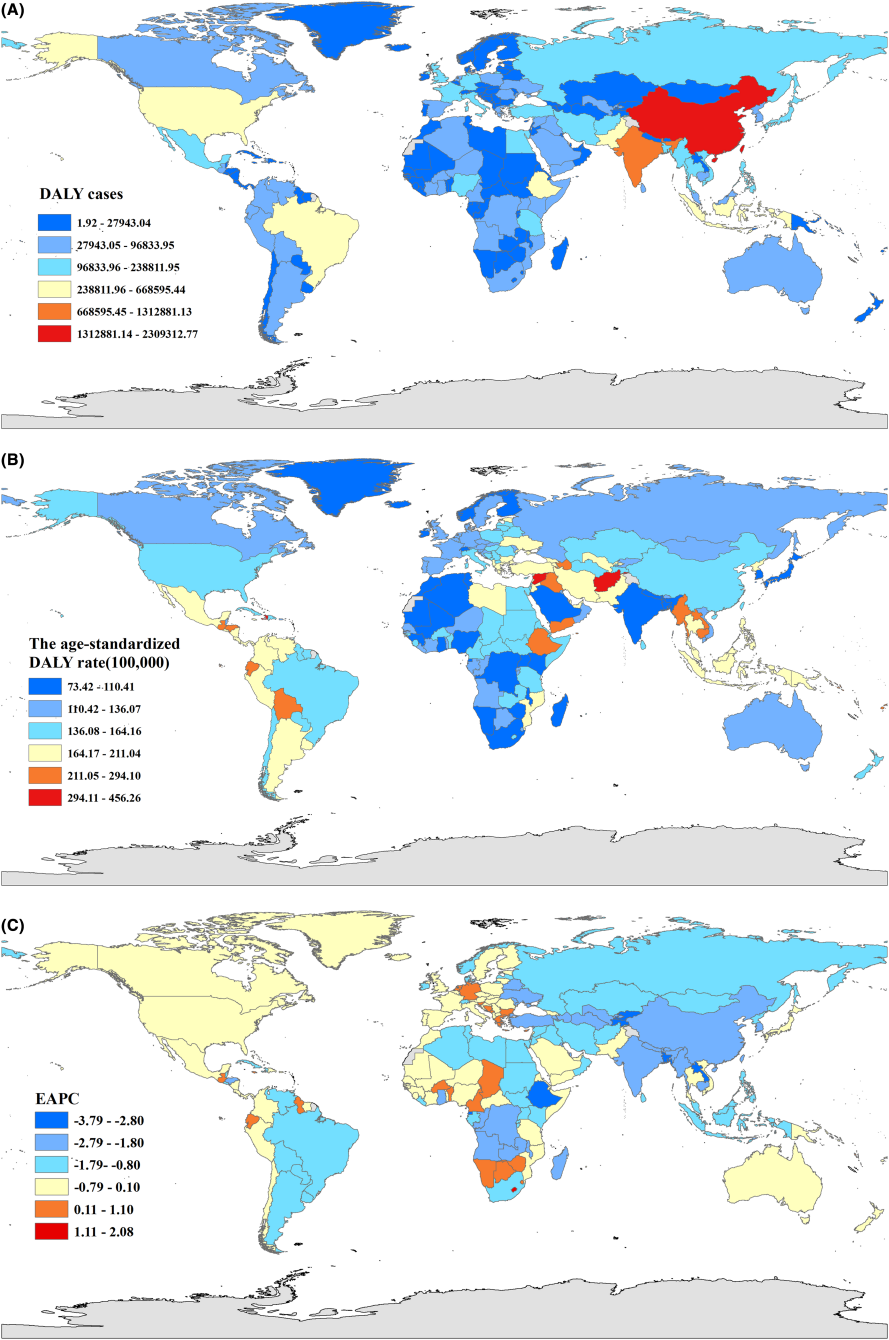
The global disease burden of Leukemia for both sexes in 204 countries and territories. (A) The number of DALY cases of Leukemia in 2019; (B) The age‐standardized DALY rate of Leukemia in 2019; (C) The EAPC of the age‐standardized DALY rate of Leukemia from 1990 to 2019. DALY, disability‐adjusted life years; EAPC, estimated annual percentage change.

Among 204 countries and territories, China, India, and United States of America had the highest DALYs for leukemia (39.0 × 10^5^, 13.2 × 10^5^, 5.7 × 10^5^ in 1990; 23.0 × 10^5^, 13.1 × 10^5^, 6.7 × 10^5^ in 2019, respectively). The age‐specific DALY rate for leukemia between 1990 and 2019 varied across 204 countries and territories, with the Syrian Arab Republic (701.6, 95%UI 533.1 to 877.2), Ethiopia (630.9, 95%UI 318.9 to 1268.2), and Afghanistan (464.5, 95%UI 225.2 to 752.0) having the highest age‐specific DALYs for leukemia in 1990; the Syrian Arab Republic (456.3, 95%UI 348.9 to 606.3), Afghanistan (365.5, 95%UI 241.8 to 546.4), and Haiti (294.1, 95%UI 168.5 to 475.8) having the highest age‐specific DALY rates for leukemia in 2019. (Figure 5). Figure 3B demonstrates the proportion of the age‐standardized DALY rate for various leukemia subtypes at the global and regional levels in 1990 and 2019.

### 3.3 The Age, period, and cohort effects on incidence and DALY using age‐period‐cohort analysis

#### 3.3.1 Age effect

**FIGURE 6:**
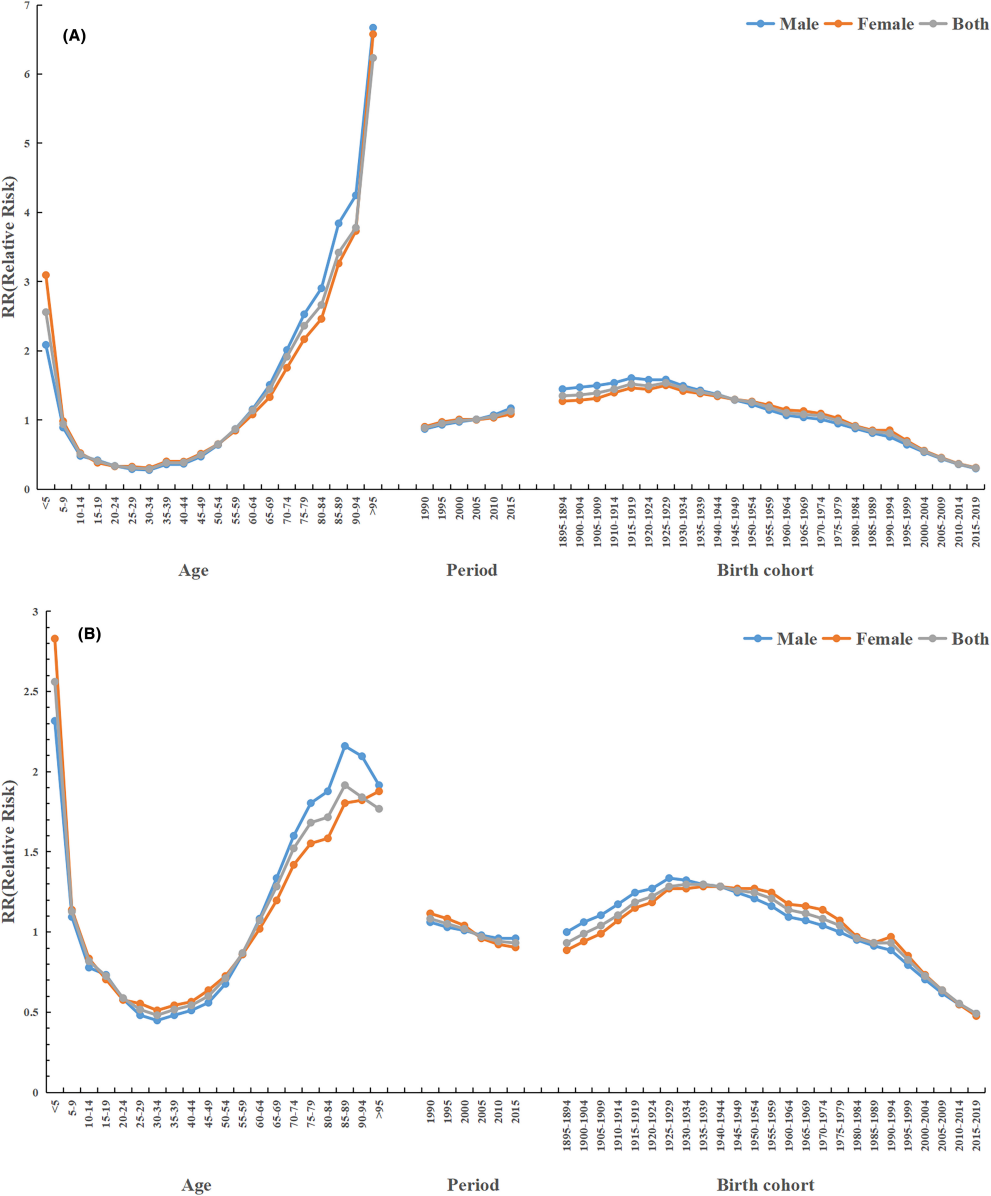
The model diagram of Age‐Period‐Cohort effect estimates for global leukemia. (A) Age‐period‐cohort analysis of Incidence; (B) Age‐period‐cohort analysis of DALY rate. To avoid the involvement of multiple dimensions of influencing factors in the disease progression process, we employed the APC model to decompose the disease progression into multiple dimensions. By analyzing the model, we obtained the true effects of the leukemia. Each age group is represented by different cohorts in different periods. The age distribution of each period is a function of age and period. APC, Age‐Period‐Cohort; DALY, Disability‐adjusted life years.

After ensuring that the period and cohort effects are constant, the age effects of global leukemia incidence and DALY rate show a “U”‐shaped distribution, with a turning point at age 30, where the relative risk of leukemia decreases with age until age 30 and, in contrast, increases with age. The age effect of leukemia showed a significant change in incidence and DALY rate with increasing age. The RR for incidence and DALY rate decreased by 88.6% and 81.2% from <5 years to 30–34 years age group, respectively, and increased by 2032.7% and 266.9% from 30 to 34 years to >95 years age group, separately (Tables 3 and 4, Figure 6A,B).

**TABLE 3:** Age‐period‐cohort model analysis of incidence.

Group	Male				Female				Both			
	Coef.	Z	*p*	RR (95% CI)	Coef.	Z	*p*	RR (95% CI)	Coef.	Z	*p*	RR (95% CI)
Age												
<5	0.74 (0.44, 1.03)	4.83	<0.001	2.09 (1.55, 2.81)	1.13 (0.83, 1.43)	7.50	<0.001	3.10 (2.30, 4.16)	0.94 (0.64, 1.24)	6.24	<0.001	2.56 (1.90, 3.46)
5–9	−0.11 (−0.45, 0.23)	−0.66	0.510	0.89 (0.63, 1.25)	−0.02 (−0.37, 0.34)	−0.09	0.932	0.98 (0.69, 1.41)	−0.06 (−0.41, 0.29)	−0.33	0.74	0.94 (0.66, 1.34)
10–14	−0.73 (−1.13, −0.33)	−3.57	<0.001	0.48 (0.32, 0.72)	−0.65 (−1.07, −0.23)	−3.03	0.002	0.52 (0.34, 0.80)	−0.68 (−1.09, −0.27)	−3.27	0.001	0.51 (0.34, 0.76)
15–19	−0.87 (−1.27, −0.47)	−4.25	<0.001	0.42 (0.28, 0.63)	−0.96 (−1.41, −0.51)	−4.18	<0.001	0.38 (0.24, 0.60)	−0.90 (−1.32, −0.48)	−4.17	<0.001	0.41 (0.27, 0.62)
20–24	−1.09 (−1.51, −0.67)	−5.05	<0.001	0.34 (0.22, 0.51)	−1.11 (−1.57, −0.65)	−4.72	<0.001	0.33 (0.21, 0.52)	−1.09 (−1.53, −0.65)	−4.86	<0.001	0.34 (0.22, 0.52)
25–29	−1.23 (−1.67, −0.80)	−5.51	<0.001	0.29 (0.19, 0.45)	−1.12 (−1.57, −0.67)	−4.86	<0.001	0.33 (0.21, 0.51)	−1.17 (−1.62, −0.73)	−5.16	<0.001	0.31 (0.20, 0.48)
30–34	−1.28 (−1.73, −0.84)	−5.64	<0.001	0.28 (0.18, 0.43)	−1.18 (−1.65, −0.72)	−4.98	<0.001	0.31 (0.19, 0.49)	−1.23 (−1.68, −0.77)	−5.28	<0.001	0.29 (0.19, 0.46)
35–39	−1.02 (−1.42, −0.63)	−5.04	<0.001	0.36 (0.24, 0.54)	−0.91 (−1.33, −0.50)	−4.30	<0.001	0.40 (0.27, 0.61)	−0.96 (−1.37, −0.56)	−4.64	<0.001	0.38 (0.25, 0.57)
40–44	−1.00 (−1.39, −0.61)	−5.04	<0.001	0.37 (0.25, 0.54)	−0.92 (−1.33, −0.51)	−4.39	<0.001	0.40 (0.27, 0.60)	−0.95 (−1.35, −0.55)	−4.67	<0.001	0.39 (0.26, 0.58)
45–49	−0.75 (−1.10, −0.40)	−4.23	<0.001	0.47 (0.33, 0.67)	−0.67 (−1.03, −0.30)	−3.55	<0.001	0.51 (0.36, 0.74)	−0.70 (−1.06, −0.34)	−3.85	<0.001	0.50 (0.35, 0.71)
50–54	−0.44 (−0.75, −0.14)	−2.87	0.004	0.64 (0.47, 0.87)	−0.43 (−0.76, −0.10)	−2.55	0.011	0.65 (0.47, 0.91)	−0.43 (−0.74, −0.11)	−2.65	0.008	0.65 (0.48, 0.90)
55–59	−0.14 (−0.40, 0.12)	−1.04	0.297	0.87 (0.67, 1.13)	−0.17 (−0.45, 0.12)	−1.13	0.258	0.85 (0.63, 1.13)	−0.14 (−0.41, 0.13)	−1.00	0.316	0.87 (0.66, 1.14)
60–64	0.14 (−0.08, 0.37)	1.28	0.200	1.16 (0.93, 1.44)	0.08 (−0.17, 0.33)	0.61	0.542	1.08 (0.84, 1.39)	0.13 (−0.11, 0.36)	1.05	0.293	1.14 (0.90, 1.43)
65–69	0.41 (0.22, 0.60)	4.30	<0.001	1.51 (1.25, 1.82)	0.29 (0.07, 0.50)	2.57	0.010	1.33 (1.07, 1.66)	0.37 (0.16, 0.57)	3.56	<0.001	1.45 (1.17, 1.77)
70–74	0.70 (0.54, 0.86)	8.72	<0.001	2.01 (1.72, 2.36)	0.56 (0.38, 0.75)	5.92	<0.001	1.76 (1.46, 2.12)	0.65 (0.47, 0.82)	7.40	<0.001	1.92 (1.60, 2.27)
75–79	0.93 (0.79, 1.07)	13.09	<0.001	2.53 (2.20, 2.91)	0.77 (0.61, 0.94)	9.09	<0.001	2.17 (1.84, 2.56)	0.86 (0.70, 1.01)	11.02	<0.001	2.36 (2.01, 2.75)
80–84	1.07 (0.93, 1.20)	15.45	<0.001	2.90 (2.54, 3.33)	0.90 (0.74, 1.06)	10.87	<0.001	2.46 (2.09, 2.90)	0.98 (0.83, 1.12)	12.82	<0.001	2.66 (2.29, 3.06)
85–89	1.35 (1.21, 1.49)	18.94	<0.001	3.84 (3.34, 4.42)	1.18 (1.02, 1.35)	14.17	<0.001	3.26 (2.77, 3.84)	1.23 (1.08, 1.39)	15.88	<0.001	3.42 (2.94, 4.01)
90–94	1.45 (1.29, 1.60)	17.89	<0.001	4.25 (3.63, 4.98)	1.32 (1.14, 1.50)	14.26	<0.001	3.73 (3.11, 4.47)	1.33 (1.16, 1.50)	15.22	<0.001	3.78 (3.19, 4.48)
>95	1.90 (1.72, 2.08)	20.55	<0.001	6.67 (5.57, 7.99)	1.88 (1.68, 2.08)	18.45	<0.001	6.58 (5.38, 8.03)	1.83 (1.64, 2.03)	18.79	<0.001	6.23 (5.16, 7.61)
Period												
1990	−0.14 (−0.26, −0.02)	−2.27	0.023	0.87 (0.77, 0.98)	−0.1 (−0.23, 0.03)	−1.49	0.135	0.90 (0.79, 1.03)	−0.12 (−0.25, 0.00)	−1.89	0.059	0.89 (0.78, 1.00)
1995	−0.07 (−0.17, 0.02)	−1.45	0.146	0.93 (0.85, 1.02)	−0.03 (−0.14, 0.08)	−0.49	0.625	0.97 (0.87, 1.09)	−0.05 (−0.15, 0.05)	−0.95	0.342	0.95 (0.86, 1.05)
2000	−0.03 (−0.11, 0.06)	−0.63	0.527	0.97 (0.90, 1.06)	0.01 (−0.09, 0.11)	0.18	0.860	1.01 (0.92, 1.11)	−0.01 (−0.1, 0.08)	−0.18	0.858	0.99 (0.90, 1.08)
2005	0.01 (−0.07, 0.09)	0.22	0.828	1.01 (0.93, 1.09)	0.00 (−0.09, 0.10)	0.07	0.944	1.00 (0.91, 1.11)	0.01 (−0.08, 0.10)	0.19	0.850	1.01 (0.92, 1.11)
2010	0.07 (−0.02, 0.16)	1.47	0.143	1.07 (0.98, 1.17)	0.03 (−0.07, 0.14)	0.60	0.547	1.03 (0.93, 1.15)	0.05 (−0.05, 0.15)	1.04	0.299	1.05 (0.95, 1.16)
2015	0.16 (0.05, 0.27)	2.76	0.006	1.17 (1.05, 1.31)	0.08 (−0.04, 0.21)	1.30	0.194	1.09 (0.96, 1.23)	0.12 (0.00, 0.24)	1.95	0.051	1.13 (1.00, 1.27)
Birth cohort												
1895–1899	0.37 (0.08, 0.66)	2.49	0.013	1.45 (1.08, 1.94)	0.24 (−0.08, 0.57)	1.46	0.146	1.27 (0.92, 1.76)	0.30 (−0.02, 0.61)	1.86	0.062	1.35 (0.98, 1.84)
1900–1904	0.39 (0.16, 0.62)	3.28	0.001	1.47 (1.17, 1.86)	0.25 (−0.01, 0.51)	1.90	0.058	1.29 (0.99, 1.67)	0.31 (0.06, 0.56)	2.43	0.015	1.36 (1.06, 1.75)
1905–1909	0.40 (0.21, 0.60)	4.17	<0.001	1.50 (1.24, 1.81)	0.27 (0.06, 0.49)	2.47	0.013	1.31 (1.06, 1.63)	0.33 (0.12, 0.53)	3.13	0.002	1.39 (1.13, 1.70)
1910–1914	0.43 (0.27, 0.59)	5.35	<0.001	1.54 (1.31, 1.80)	0.33 (0.15, 0.52)	3.60	<0.001	1.40 (1.16, 1.67)	0.37 (0.20, 0.54)	4.26	<0.001	1.45 (1.22, 1.72)
1915–1919	0.47 (0.34, 0.60)	7.16	<0.001	1.61 (1.41, 1.83)	0.38 (0.23, 0.54)	4.90	<0.001	1.47 (1.26, 1.71)	0.42 (0.27, 0.56)	5.71	<0.001	1.52 (1.31, 1.75)
1920–1924	0.46 (0.35, 0.57)	8.21	<0.001	1.58 (1.42, 1.77)	0.37 (0.23, 0.50)	5.43	<0.001	1.44 (1.26, 1.65)	0.40 (0.28, 0.52)	6.42	<0.001	1.49 (1.32, 1.68)
1925–1929	0.46 (0.34, 0.58)	7.52	<0.001	1.59 (1.41, 1.79)	0.41 (0.26, 0.55)	5.42	<0.001	1.50 (1.30, 1.74)	0.43 (0.29, 0.56)	6.20	<0.001	1.54 (1.34, 1.75)
1930–1934	0.40 (0.26, 0.54)	5.56	<0.001	1.50 (1.30, 1.72)	0.35 (0.18, 0.52)	4.00	<0.001	1.42 (1.20, 1.69)	0.38 (0.22, 0.53)	4.72	<0.001	1.46 (1.25, 1.70)
1935–1939	0.36 (0.18, 0.53)	4.06	<0.001	1.43 (1.20, 1.70)	0.32 (0.12, 0.53)	3.12	0.002	1.38 (1.13, 1.70)	0.34 (0.16, 0.53)	3.60	<0.001	1.40 (1.17, 1.70)
1940–1944	0.32 (0.11, 0.52)	3.00	0.003	1.37 (1.12, 1.68)	0.29 (0.06, 0.53)	2.42	0.015	1.34 (1.06, 1.70)	0.31 (0.09, 0.53)	2.73	0.006	1.36 (1.09, 1.70)
1945–1949	0.25 (0.01, 0.50)	2.04	0.042	1.29 (1.01, 1.65)	0.26 (−0.02, 0.54)	1.83	0.067	1.30 (0.98, 1.71)	0.26 (0.00, 0.52)	1.97	0.049	1.30 (1.00, 1.68)
1950–1954	0.21 (−0.08, 0.49)	1.40	0.161	1.23 (0.92, 1.64)	0.24 (−0.08, 0.56)	1.46	0.143	1.27 (0.92, 1.74)	0.23 (−0.08, 0.53)	1.46	0.143	1.26 (0.92, 1.70)
1955–1959	0.14 (−0.19, 0.46)	0.81	0.418	1.15 (0.82, 1.59)	0.20 (−0.16, 0.55)	1.07	0.284	1.22 (0.85, 1.74)	0.17 (−0.17, 0.51)	0.96	0.335	1.19 (0.84, 1.67)
1960–1964	0.07 (−0.3, 0.44)	0.35	0.726	1.07 (0.74, 1.55)	0.14 (−0.26, 0.53)	0.67	0.504	1.15 (0.77, 1.70)	0.10 (−0.28, 0.49)	0.53	0.593	1.11 (0.76, 1.63)
1965–1969	0.04 (−0.37, 0.44)	0.19	0.852	1.04 (0.69, 1.56)	0.12 (−0.30, 0.55)	0.57	0.571	1.13 (0.74, 1.73)	0.08 (−0.33, 0.50)	0.40	0.690	1.08 (0.72, 1.65)
1970–1974	0.01 (−0.41, 0.43)	0.05	0.957	1.01 (0.66, 1.54)	0.09 (−0.35, 0.53)	0.40	0.691	1.09 (0.70, 1.71)	0.06 (−0.38, 0.49)	0.25	0.803	1.06 (0.68, 1.63)
1975–1979	−0.05 (−0.48, 0.38)	−0.24	0.808	0.95 (0.62, 1.46)	0.02 (−0.43, 0.48)	0.11	0.915	1.03 (0.65, 1.62)	−0.01 (−0.45, 0.43)	−0.04	0.964	0.99 (0.64, 1.54)
1980–1984	−0.13 (−0.55, 0.29)	−0.60	0.546	0.88 (0.58, 1.34)	−0.09 (−0.54, 0.37)	−0.38	0.707	0.92 (0.58, 1.44)	−0.10 (−0.54, 0.33)	−0.46	0.643	0.90 (0.58, 1.39)
1985–1989	−0.21 (−0.6, 0.18)	−1.04	0.299	0.81 (0.55, 1.20)	−0.16 (−0.58, 0.26)	−0.75	0.451	0.85 (0.56, 1.29)	−0.18 (−0.58, 0.23)	−0.87	0.386	0.84 (0.56, 1.26)
1990–1994	−0.28 (−0.61, 0.05)	−1.64	0.101	0.76 (0.55, 1.05)	−0.16 (−0.49, 0.17)	−0.95	0.341	0.85 (0.62, 1.18)	−0.21 (−0.54, 0.12)	−1.26	0.207	0.81 (0.58, 1.13)
1995–1999	−0.44 (−0.79, −0.09)	−2.45	0.014	0.64 (0.45, 0.92)	−0.36 (−0.71, 0.00)	−1.98	0.047	0.70 (0.49, 1.00)	−0.39 (−0.74, −0.04)	−2.19	0.029	0.68 (0.48, 0.96)
2000–2004	−0.62 (−1.01, −0.24)	−3.16	0.002	0.54 (0.36, 0.79)	−0.58 (−0.97, −0.20)	−2.95	0.003	0.56 (0.38, 0.82)	−0.60 (−0.98, −0.21)	−3.03	0.002	0.55 (0.38, 0.81)
2005–2009	−0.82 (−1.25, −0.38)	−3.70	<0.001	0.44 (0.29, 0.68)	−0.78 (−1.21, −0.35)	−3.57	<0.001	0.46 (0.30, 0.70)	−0.80 (−1.23, −0.37)	−3.62	<0.001	0.45 (0.29, 0.69)
2010–2014	−1.02 (−1.51, −0.53)	−4.05	<0.001	0.36 (0.22, 0.59)	−1.00 (−1.49, −0.51)	−4.01	<0.001	0.37 (0.23, 0.60)	−1.01 (−1.50, −0.52)	−4.02	<0.001	0.36 (0.22, 0.59)
2015–2019	−1.20 (−1.87, −0.53)	−3.52	<0.001	0.30 (0.15, 0.59)	−1.16 (−1.80, −0.53)	−3.59	<0.001	0.31 (0.17, 0.59)	−1.18 (−1.83, −0.53)	−3.55	<0.001	0.31 (0.16, 0.59)
AIC	5.29				5.06				5.16			
BIC	−342.64				−343.92				−343.82			
df	72				72				72			
Deviance	2.06				0.78				0.88			

Abbreviations: AIC, Akaike Information Criterion; BIC, Bayesian Information Criterion; CI, confidence interval; RR, relative risk.

**TABLE 4:** Age‐period‐cohort model analysis of DALY.

Group	Male				Female				Both			
	Coef.	Z	*p*	RR (95% CI)	Coef.	Z	*p*	RR (95% CI)	Coef.	Z	*p*	RR (95% CI)
Age												
<5	0.84 (0.79, 0.89)	30.5	<0.001	2.32 (2.20, 2.44)	1.04 (0.98, 1.10)	35.11	<0.001	2.83 (2.66, 3.00)	0.94 (0.89, 1.00)	33.06	<0.001	2.56 (2.44, 2.72)
5–9	0.09 (0.03, 0.15)	3.03	0.002	1.09 (1.03, 1.16)	0.13 (0.07, 0.20)	3.88	<0.001	1.14 (1.07, 1.22)	0.12 (0.06, 0.19)	3.83	<0.001	1.13 (1.06, 1.21)
10–14	−0.25 (−0.31, −0.18)	−7.38	<0.001	0.78 (0.73, 0.84)	−0.18 (−0.25, −0.11)	−4.92	<0.001	0.84 (0.78, 0.90)	−0.20 (−0.27, −0.14)	−5.84	<0.001	0.82 (0.76, 0.87)
15–19	−0.31 (−0.38, −0.25)	−9.37	<0.001	0.73 (0.68, 0.78)	−0.35 (−0.43, −0.28)	−9.30	<0.001	0.70 (0.65, 0.76)	−0.32 (−0.38, −0.25)	−8.93	<0.001	0.73 (0.68, 0.78)
20–24	−0.54 (−0.61, −0.47)	−15.22	<0.001	0.58 (0.54, 0.63)	−0.55 (−0.63, −0.47)	−13.63	<0.001	0.58 (0.53, 0.63)	−0.53 (−0.61, −0.46)	−14.12	<0.001	0.59 (0.54, 0.63)
25–29	−0.73 (−0.81, −0.66)	−19.10	<0.001	0.48 (0.44, 0.52)	−0.59 (−0.67, −0.51)	−14.6	<0.001	0.55 (0.51, 0.60)	−0.66 (−0.73, −0.58)	−16.65	<0.001	0.52 (0.48, 0.56)
30–34	−0.8 (−0.88, −0.72)	−20.29	<0.001	0.45 (0.41, 0.49)	−0.67 (−0.75, −0.59)	−15.82	<0.001	0.51 (0.47, 0.55)	−0.73 (−0.81, −0.65)	−17.84	<0.001	0.48 (0.44, 0.52)
35–39	−0.73 (−0.8, −0.65)	−19.07	<0.001	0.48 (0.45, 0.52)	−0.61 (−0.69, −0.53)	−14.85	<0.001	0.54 (0.50, 0.59)	−0.66 (−0.74, −0.59)	−16.72	<0.001	0.52 (0.48, 0.55)
40–44	−0.67 (−0.74, −0.60)	−18.03	<0.001	0.51 (0.48, 0.55)	−0.57 (−0.65, −0.49)	−14.22	<0.001	0.57 (0.52, 0.61)	−0.61 (−0.69, −0.54)	−15.85	<0.001	0.54 (0.50, 0.58)
45–49	−0.58 (−0.65, −0.51)	−16.4	<0.001	0.56 (0.52, 0.60)	−0.45 (−0.53, −0.38)	−11.82	<0.001	0.64 (0.59, 0.68)	−0.51 (−0.58, −0.44)	−13.85	<0.001	0.60 (0.56, 0.64)
50–54	−0.39 (−0.45, −0.32)	−11.95	<0.001	0.68 (0.64, 0.73)	−0.32 (−0.39, −0.25)	−8.88	<0.001	0.73 (0.68, 0.78)	−0.34 (−0.41, −0.27)	−10.06	<0.001	0.71 (0.66, 0.76)
55–59	−0.15 (−0.21, −0.09)	−5.22	<0.001	0.86 (0.81, 0.91)	−0.15 (−0.21, −0.09)	−4.55	<0.001	0.86 (0.81, 0.91)	−0.14 (−0.2, −0.08)	−4.43	<0.001	0.87 (0.82, 0.92)
60–64	0.08 (0.03, 0.13)	2.99	0.003	1.08 (1.03, 1.14)	0.02 (−0.04, 0.08)	0.72	0.469	1.02 (0.96, 1.08)	0.07 (0.01, 0.12)	2.40	0.016	1.07 (1.01, 1.13)
65–69	0.29 (0.25, 0.33)	12.90	<0.001	1.34 (1.28, 1.39)	0.18 (0.12, 0.23)	6.52	<0.001	1.20 (1.13, 1.26)	0.25 (0.21, 0.30)	10.28	<0.001	1.28 (1.23, 1.35)
70–74	0.47 (0.43, 0.51)	23.17	<0.001	1.60 (1.54, 1.67)	0.35 (0.30, 0.40)	14.05	<0.001	1.42 (1.35, 1.49)	0.42 (0.38, 0.47)	18.92	<0.001	1.52 (1.46, 1.60)
75–79	0.59 (0.55, 0.63)	31.41	<0.001	1.80 (1.73, 1.88)	0.44 (0.40, 0.49)	18.75	<0.001	1.55 (1.49, 1.63)	0.52 (0.48, 0.56)	24.76	<0.001	1.68 (1.62, 1.75)
80–84	0.63 (0.59, 0.67)	33.71	<0.001	1.88 (1.80, 1.95)	0.46 (0.42, 0.51)	19.41	<0.001	1.58 (1.52, 1.67)	0.54 (0.49, 0.58)	25.21	<0.001	1.72 (1.63, 1.79)
85–89	0.77 (0.74, 0.81)	41.16	<0.001	2.16 (2.10, 2.25)	0.59 (0.54, 0.64)	24.77	<0.001	1.80 (1.72, 1.90)	0.65 (0.60, 0.69)	30.08	<0.001	1.92 (1.82, 1.99)
90–94	0.74 (0.69, 0.78)	35.71	<0.001	2.10 (1.99, 2.18)	0.60 (0.55, 0.65)	23.52	<0.001	1.82 (1.73, 1.92)	0.61 (0.56, 0.65)	25.84	<0.001	1.84 (1.75, 1.92)
>95	0.65 (0.60, 0.70)	27.07	<0.001	1.92 (1.82, 2.01)	0.63 (0.57, 0.68)	21.65	<0.001	1.88 (1.77, 1.97)	0.57 (0.52, 0.62)	21.14	<0.001	1.77 (1.68, 1.86)
Period												
1990	0.06 (0.04, 0.09)	4.74	<0.001	1.06 (1.04, 1.09)	0.11 (0.08, 0.13)	7.25	<0.001	1.12 (1.08, 1.14)	0.08 (0.06, 0.11)	6.00	<0.001	1.08 (1.06, 1.12)
1995	0.03 (0.01, 0.06)	2.83	0.005	1.03 (1.01, 1.06)	0.08 (0.05, 0.11)	5.65	<0.001	1.08 (1.05, 1.12)	0.05 (0.03, 0.08)	4.21	<0.001	1.05 (1.03, 1.08)
2000	0.01 (−0.02, 0.03)	0.60	0.549	1.01 (0.98, 1.03)	0.04 (0.01, 0.07)	2.95	0.003	1.04 (1.01, 1.07)	0.02 (0.00, 0.05)	1.78	0.074	1.02 (1.00, 1.05)
2005	−0.02 (−0.04, 0.00)	−1.85	0.065	0.98 (0.96, 1.00)	−0.04 (−0.07, −0.01)	−2.77	0.006	0.96 (0.93, 0.99)	−0.03 (−0.05, 0.00)	−2.25	0.024	0.97 (0.95, 1.00)
2010	−0.04 (−0.07, −0.02)	−3.37	0.001	0.96 (0.93, 0.98)	−0.08 (−0.11, −0.05)	−5.50	<0.001	0.92 (0.90, 0.95)	−0.06 (−0.09, −0.03)	−4.38	<0.001	0.94 (0.91, 0.97)
2015	−0.04 (−0.06, −0.01)	−2.87	0.004	0.96 (0.94, 0.99)	−0.10 (−0.14, −0.07)	−6.57	<0.001	0.90 (0.87, 0.93)	−0.07 (−0.10, −0.04)	−4.85	<0.001	0.93 (0.90, 0.96)
Birth cohort												
1895–1899	0.00 (−0.09, 0.10)	0.03	0.974	1.00 (0.91, 1.11)	−0.12 (−0.23, −0.01)	−2.06	0.040	0.89 (0.79, 0.99)	−0.07 (−0.18, 0.04)	−1.28	0.200	0.93 (0.84, 1.04)
1900–1904	0.06 (0.00, 0.13)	1.90	0.057	1.06 (1.00, 1.14)	−0.06 (−0.14, 0.02)	−1.44	0.150	0.94 (0.87, 1.02)	−0.01 (−0.09, 0.06)	−0.29	0.773	0.99 (0.91, 1.06)
1905–1909	0.10 (0.05, 0.16)	3.86	<0.001	1.11 (1.05, 1.17)	−0.01 (−0.07, 0.06)	−0.19	0.852	0.99 (0.93, 1.06)	0.04 (−0.02, 0.10)	1.22	0.221	1.04 (0.98, 1.11)
1910–1914	0.16 (0.11, 0.20)	6.88	<0.001	1.17 (1.12, 1.22)	0.07 (0.02, 0.13)	2.51	0.012	1.07 (1.02, 1.14)	0.10 (0.05, 0.15)	3.94	<0.001	1.11 (1.05, 1.16)
1915–1919	0.22 (0.18, 0.26)	11.18	<0.001	1.25 (1.20, 1.30)	0.14 (0.09, 0.19)	5.68	<0.001	1.15 (1.09, 1.21)	0.17 (0.13, 0.21)	7.46	<0.001	1.19 (1.14, 1.23)
1920–1924	0.24 (0.21, 0.28)	13.56	<0.001	1.27 (1.23, 1.32)	0.17 (0.12, 0.21)	7.42	<0.001	1.19 (1.13, 1.23)	0.20 (0.16, 0.24)	9.53	<0.001	1.22 (1.17, 1.27)
1925–1929	0.29 (0.25, 0.32)	15.75	<0.001	1.34 (1.28, 1.38)	0.24 (0.19, 0.28)	10.05	<0.001	1.27 (1.21, 1.32)	0.25 (0.21, 0.30)	12.11	<0.001	1.28 (1.23, 1.35)
1930–1934	0.28 (0.24, 0.32)	13.77	<0.001	1.32 (1.27, 1.38)	0.24 (0.19, 0.29)	9.62	<0.001	1.27 (1.21, 1.34)	0.26 (0.22, 0.30)	11.51	<0.001	1.30 (1.25, 1.35)
1935–1939	0.26 (0.22, 0.31)	11.70	<0.001	1.30 (1.25, 1.36)	0.25 (0.20, 0.31)	9.19	<0.001	1.28 (1.22, 1.36)	0.26 (0.21, 0.31)	10.48	<0.001	1.30 (1.23, 1.36)
1940–1944	0.25 (0.20, 0.30)	9.85	<0.001	1.28 (1.22, 1.35)	0.25 (0.19, 0.31)	8.32	<0.001	1.28 (1.21, 1.36)	0.25 (0.20, 0.31)	9.15	<0.001	1.28 (1.22, 1.36)
1945–1949	0.22 (0.17, 0.28)	7.84	<0.001	1.25 (1.19, 1.32)	0.24 (0.18, 0.31)	7.27	<0.001	1.27 (1.20, 1.36)	0.23 (0.17, 0.29)	7.69	<0.001	1.26 (1.19, 1.34)
1950–1954	0.19 (0.13, 0.26)	6.14	<0.001	1.21 (1.14, 1.30)	0.24 (0.17, 0.31)	6.62	<0.001	1.27 (1.19, 1.36)	0.22 (0.15, 0.28)	6.50	<0.001	1.25 (1.16, 1.32)
1955–1959	0.15 (0.08, 0.22)	4.38	<0.001	1.16 (1.08, 1.25)	0.22 (0.15, 0.30)	5.73	<0.001	1.25 (1.16, 1.35)	0.19 (0.12, 0.26)	5.13	<0.001	1.21 (1.13, 1.30)
1960–1964	0.09 (0.02, 0.17)	2.48	0.013	1.09 (1.02, 1.19)	0.16 (0.07, 0.24)	3.76	<0.001	1.17 (1.07, 1.27)	0.13 (0.05, 0.20)	3.21	0.001	1.14 (1.05, 1.22)
1965–1969	0.07 (−0.01, 0.14)	1.70	0.090	1.07 (0.99, 1.15)	0.15 (0.06, 0.23)	3.48	0.001	1.16 (1.06, 1.26)	0.11 (0.03, 0.19)	2.66	0.008	1.12 (1.03, 1.21)
1970–1974	0.04 (−0.04, 0.12)	1.04	0.300	1.04 (0.96, 1.13)	0.13 (0.04, 0.21)	2.97	0.003	1.14 (1.04, 1.23)	0.08 (0.00, 0.16)	2.08	0.038	1.08 (1.00, 1.17)
1975–1979	0.00 (−0.07, 0.08)	0.03	0.977	1.00 (0.93, 1.08)	0.07 (−0.01, 0.15)	1.66	0.098	1.07 (0.99, 1.16)	0.04 (−0.04, 0.11)	0.93	0.353	1.04 (0.96, 1.12)
1980–1984	−0.05 (−0.12, 0.02)	−1.44	0.150	0.95 (0.89, 1.02)	−0.03 (−0.11, 0.05)	−0.75	0.454	0.97 (0.90, 1.05)	−0.04 (−0.11, 0.04)	−0.97	0.334	0.96 (0.90, 1.04)
1985–1989	−0.09 (−0.16, −0.03)	−2.77	0.006	0.91 (0.85, 0.97)	−0.07 (−0.14, 0.01)	−1.74	0.081	0.93 (0.87, 1.01)	−0.07 (−0.14, 0.00)	−2.10	0.036	0.93 (0.87, 1.00)
1990–1994	−0.12 (−0.18, −0.07)	−4.26	<0.001	0.89 (0.84, 0.93)	−0.03 (−0.09, 0.03)	−1.01	0.311	0.97 (0.91, 1.03)	−0.07 (−0.13, −0.02)	−2.47	0.014	0.93 (0.88, 0.98)
1995–1999	−0.23 (−0.29, −0.17)	−7.31	<0.001	0.79 (0.75, 0.84)	−0.16 (−0.22, −0.09)	−4.54	<0.001	0.85 (0.80, 0.91)	−0.19 (−0.25, −0.12)	−5.72	<0.001	0.83 (0.78, 0.89)
2000–2004	−0.35 (−0.42, −0.28)	−9.97	<0.001	0.70 (0.66, 0.76)	−0.31 (−0.38, −0.23)	−8.00	<0.001	0.73 (0.68, 0.79)	−0.32 (−0.39, −0.25)	−8.77	<0.001	0.73 (0.68, 0.78)
2005–2009	−0.48 (−0.55, −0.40)	−11.90	<0.001	0.62 (0.58, 0.67)	−0.45 (−0.53, −0.36)	−10.11	<0.001	0.64 (0.59, 0.70)	−0.45 (−0.53, −0.37)	−10.82	<0.001	0.64 (0.59, 0.69)
2010–2014	−0.60 (−0.69, −0.51)	−12.93	<0.001	0.55 (0.50, 0.60)	−0.60 (−0.70, −0.50)	−11.6	<0.001	0.55 (0.50, 0.61)	−0.59 (−0.69, −0.5)	−12.08	<0.001	0.55 (0.50, 0.61)
2015–2019	−0.71 (−0.84, −0.59)	−11.13	<0.001	0.49 (0.43, 0.55)	−0.74 (−0.88, −0.60)	−10.48	<0.001	0.48 (0.41, 0.55)	−0.71 (−0.84, −0.58)	−10.65	<0.001	0.49 (0.43, 0.56)
AIC	8.33				8.09				8.15			
BIC	−332.96				−325.49				−334.78			
df	72				72				72			
Deviance	11.74				19.21				9.92			

Abbreviations: AIC, Akaike Information Criterion; BIC, Bayesian Information Criterion; CI, confidence interval; DALY, disability‐adjusted life years; RR, relative risk.

#### 3.3.2 Period Effect

From 1990 to 2019, the global RR of leukemia incidence showed an increasing trend with a growth rate of 27.1%, in which the increase was greater in male (34.4%) than in female (20.3%), and our findings suggest a gradual increase in the incidence of leukemia over time (Table 3, Figure 6A).

Compared with 1990, the RR of DALY rates for leukemia in 2019 showed a decreasing trend in both males and females globally, and the decreasing rate was lower in males than in females, at the rates of 9.5% and 18.9%, respectively. The period effects indicated that the period effects of leukemia incidence and DALY rates were relatively stable (Table 4, Figure 6B).

#### 3.3.3 Cohort effect

After ensuring constant age and period effects, the overall cohort effect for leukemia showed an increasing and then decreasing trend. Morbidity and DALY rates peaked at disease risk in cohorts 1925–1929 and 1935–1939, RR = 1.54 (95%UI 1.34 to 1.75) and RR = 1.30 (95%UI 1.23 to 1.36), after which incidence risk tended to declining, with later birth cohorts having lower RR than earlier birth cohorts, except for some individual cohorts (Tables 3 and 4, Figure 6A,B).

### 3.4 Risk factors for disease burden in leukemia

**FIGURE 7:**
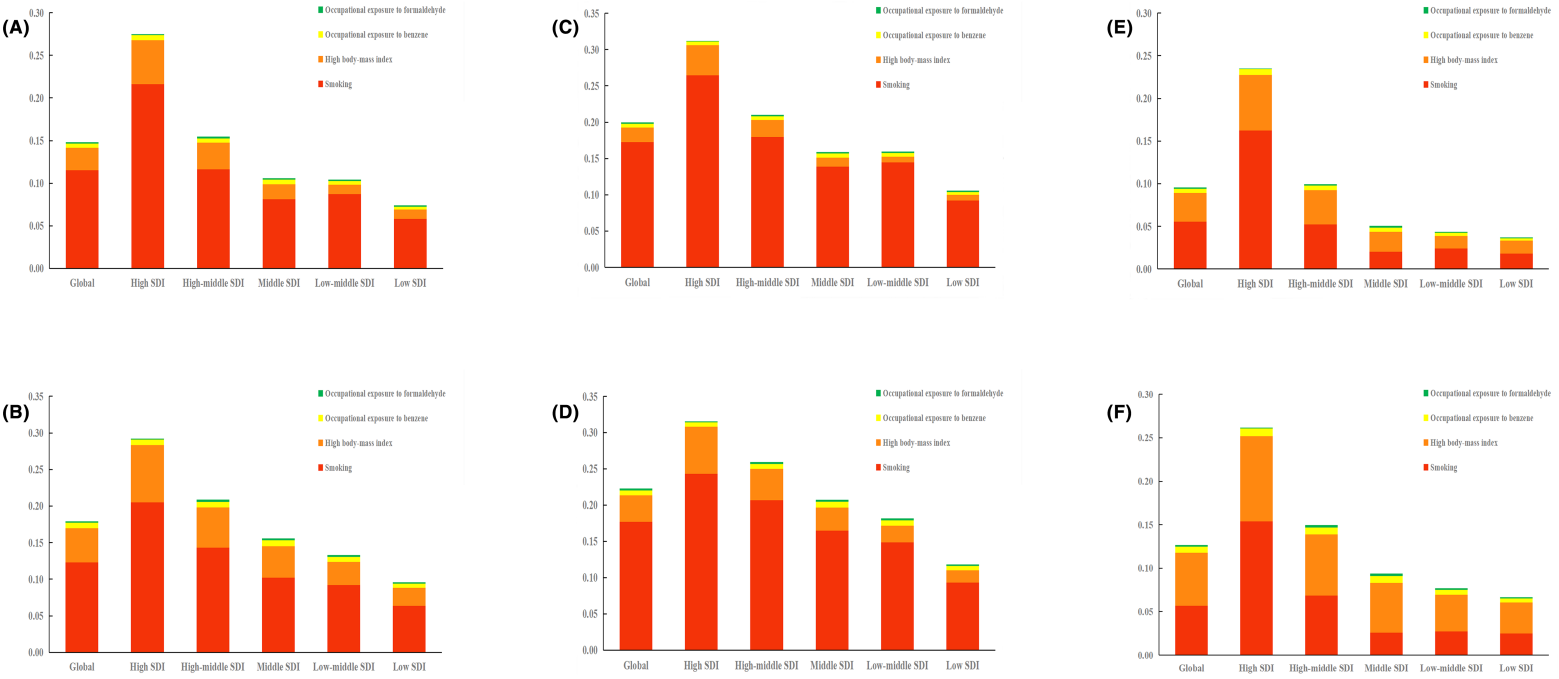
The leukemia DALYs attributable to risk factors in 1990 and 2019. In 1990 and 2019, smoking, high body‐mass index, occupational exposure to benzene and occupational exposure to formaldehyde were risk factors for leukemia DALY, especially in areas with high SDI. From 1990 to 2019, there were gender differences in the burden of leukemia disease caused by smoking and high body‐mass index worldwide. (A) Global risk factor attribution analysis in 1990, (B) global risk factor attribution analysis in 2019, (C) global risk factor attribution analysis for men in 1990, (D) global risk factor attribution analysis for men in 2019, (E) global risk factor attribution analysis for female in 1990, and (F) global risk factor attribution analysis for female in 2019. DALY, Disability‐adjusted life years.

In 1990 and 2019, smoking, high body‐mass index, occupational exposure to benzene, and occupational exposure to formaldehyde were risk factors for DALY in leukemia, especially in areas with high SDI. From 1990 to 2019, there were gender differences in the burden of leukemia disease caused by smoking and high body‐mass index worldwide. The burden of leukemia disease in males and females was attributed to smoking at 17.23% (95%UI 10.24% to 24.70%) and 5.55% (95%UI 2.92% to 8.78%) in 1990, and 17.68% (95%UI 10.85% to 24.45%) and 5.67% (95%UI 3.04% to 8.90%) in 2019, respectively, with males being higher than females; In 1990, 2.03% (95%UI 0.79% to 3.97%) and 3.38% (95%UI 1.18% to 6.86%) were attributed to high body‐mass index, while in 2019, 3.69% (95%UI 1.69% to 6.30%) and 6.08% (95%UI 2.35% to 11.10%) were attributed to high body‐mass index, with females being higher than males. This indicates that the burden of leukemia in women caused by high body‐mass index exceeds that caused by smoking (Figure 7).

## 4 DISCUSSION

A study reported that the 5‐year survival rate of leukemia patients in the United States increased from 33.2% to 66.1%. There are significant differences in the 5‐year survival rates of different subtypes of leukemia. Compared with 1975, the 5‐year survival rates for ALL, CLL, CML, and other lymphocytic leukemias were 69.5%, 89.5%, 72.8%, and 80.2%, respectively. Previous studies have shown that the incidence of leukemia in men is about 1.7 times higher than that in women, and the susceptibility of leukemia to men has been observed worldwide.^41^, ^42^ This study provides a systematic and comprehensive analysis of the epidemiological trends of leukemia at the global, regional, and national levels by calculating the EAPC for the incidence and DALY rates of leukemia from 1990 to 2019. The findings show that although the ASIR and age‐standardized DALY rates for leukemia have been on a declining trend globally over the past 30 years, new cases of leukemia and DALYs are on the rise due to population growth and population aging. Therefore, it remains necessary to analyze the patterns of leukemia incidence and DALY and to explore the etiology, natural history, and differences behind these changes in trends.

The age effect showed a “U”‐shaped distribution of incidence and DALY rates with increasing age; the period effect showed that the incidence of leukemia increased over time, but the period trend of DALY rates was decreasing; the cohort effect showed an overall decreasing trend of incidence and DALY rates, with later birth cohorts having lower incidence and DALY rates than earlier birth cohorts. The results of the present study showed that both ASIR and the age‐standardized DALY rate of leukemia were heavy in the <5 years and >60 years age groups, which is consistent with previous findings that leukemia develops in all age groups, with the highest incidence of childhood leukemia in the 0–4 years age group.^43^ In the United Kingdom,^44^ leukemia accounts for 34% of all malignancies in children under 15 years of age; the results of a Piedmont study^45^ reported that leukemia was the first cancer in children and adolescents between 1967 and 2011 and that the peak age was 1–4 years, accounting for 75.9%, consistent with the results of this study. This may be related to the incomplete development of the child's immune system, prenatal or postnatal exposure to ionizing radiation (especially X‐rays),^46^ and parental exposure to certain chemically exposed occupations,^47^ especially benzene exposure. The higher disease burden of leukemia in the >60 years age group. Previous studies have shown that with age, physical function decreases,^48^ immunity decreases, tolerance to chemotherapy toxicity is poor,^49^ and human exposure to external risk factors is also a cumulative effect with age,^50^ for example, the health effects of behaviors such as smoking are lagging and persistent; therefore, the incidence of leukemia in the elderly population is on the rise in the current social situation where population aging is progressing rapidly and the elderly population is gradually increasing.

The results of the period effect showed an increasing trend in the incidence of leukemia, which may be attributed to the improvement of early diagnosis techniques for leukemia.^1^ The results of Chen's study^51^ showed that metabolomic studies of sera from patients with new‐onset AML and healthy volunteers identified six significantly different metabolites for early diagnosis and prognostic analysis of AML. Wang's study^52^ also found that small‐molecule metabolites detected by a metabolomics approach based on hydrogen spectroscopy nuclear magnetic resonance (^1^H‐NMR) could be used for early diagnosis of leukemia and determination of leukemia severity. Cohort effects reflect changes in early‐life environments and assume that people in uniform birth cohorts have the same exposure to disease risk factors.^37^ The results of the cohort effect study on the incidence and DALY rate showed a decreasing trend from the earlier birth cohort to the later birth cohort. On the one hand, the later birth cohort not only received better education and better awareness of health and disease prevention due to economic development,^53^ but also had better nutritional conditions and living conditions,^54^ and these social factors reduced the exposure to risk factors in this population. On the other hand, it may be that with the establishment of a universal health care system, the disease can be treated in a timely manner. With the birth of the new rural cooperative medical care in China in 2003 and the basic medical insurance for urban residents in 2007, the medical insurance system has been gradually improved,^55^ and the disease burden on leukemia patients has been reduced. Effective treatment will reduce the risk of death to a certain extent, thus reducing the disease burden of leukemia.

Our study showed that the EAPC of ASIR had an increasing trend in high SDI, high–middle SDI regions, and the EAPC of the age‐standardized DALY rate had a decreasing trend in each SDI region. The disease burden of leukemia varies across SDI regions, which may be related to the large imbalance in health care resources. The higher disease burden of leukemia in high SDI regions may be related to features such as better economic and cultural conditions, widespread availability of cancer screening, and an aging population, which have elevated the diagnosis rate of leukemia. On the contrary, among the low SDI regions, the lack of economic and medical resources, the long‐term poor access to cancer screening in this region, missed diagnoses, and incomplete case reporting have led to some surveillance bias,^56^ resulting in an underestimation of leukemia incidence and mortality in the low SDI regions. However, with the rapid development of the world economy in recent years, many regions with low SDI scores have focused on improving primary care conditions and attention to tertiary prevention, and the bias due to underdiagnosis and underreporting has gradually decreased, leading to a more accurate assessment of the disease burden of leukemia in this region.^57^

As a point of interest, in the low, low–middle and middle SDI regions, including most of Asia, Oceania, and parts of sub‐Saharan Africa, we observed a smaller declining trend in leukemia, which may be attributable to the remarkable effectiveness of local health infrastructure, international cooperation, and health assistance.^58^, ^59^ For example, the primary health care system in Brazil is very important for the health of the population to provide the most effective interventions for AML patients.^60^ In China, there is a significant downward trend in the disease burden of leukemia, which is most likely associated with the development of socioeconomic and health care resources. China's new rural cooperative medical care covers about 80% of the total rural population (about 830 million people), making health care resources more accessible and affordable.^61^, ^62^ National differences and changing trends in leukemia not only reflect the effectiveness of previous prevention strategies but also indicate that newer and tailored leukemia prevention strategies have been established.^63^

Based on the GBD 2019 database, we analyzed four risk factors for leukemia, including smoking, high body‐mass index, occupational exposure to benzene, and occupational exposure to formaldehyde. Our study found that there were gender differences in the burden of leukemia disease caused by smoking and high body‐mass index worldwide from 1990 to 2019. This gender difference may be related to men being more susceptible to risk factors. Smoking has always been considered a major environmental risk factor for the development of leukemia, with a prevalence rate of five times higher in males (25.0%) than in females (5.4%).^64^, ^65^ Previous research has shown that children whose fathers smoke have a higher risk of developing leukemia.^66^ High body mass index is the number one risk factor for women, and a multicenter cohort study in Mexico in 2019 showed a high correlation between high body mass index and mortality from leukemia.^67^ This may be related to the fact that adipocytes secrete a variety of hormones and inflammatory cytokines when they are obese, leading to chronic inflammation and subsequently increasing the risk of cancer.^68^ Therefore, in order to reduce the disease burden of leukemia, it is very important to reduce the additional burden of smoking and obesity. Strategies to reduce smoking include health education, raising tobacco taxes, and advertising, and obesity reduction strategies include supporting healthy school diets, improving nutrition education, increasing taxes on unhealthy foods, and providing subsidies for healthy foods.^69^

There are some limitations in this study. First, the disease burden of leukemia may be underestimated in some developing countries due to traditional limitations of cancer detection systems, e.g., variations between coding systems that may lead to artificial differences in disease estimates.^63^ Second, the criteria for diagnosis and classification of leukemia types vary from country to country, which may lead to complications in the analysis of understanding the changing trends in certain types of leukemia.^70^ Finally, this study only analyzed the burden of leukemia disease globally, across different SDI regions and specific countries, without delving into various provinces. Therefore, it is necessary to include research data from specific provinces to comprehensively understand the disease burden of leukemia. Therefore, these differences should be considered in the development of health care resources and health care systems.

## 5 CONCLUSION

In conclusion, the study showed that ASIR and the age‐standardized DALY rate of leukemia showed a decreasing trend from 1990 to 2019, but the disease burden of leukemia was generally increasing in high and middle SDI regions, and the disease burden was higher in men than in women. Leukemia varies greatly between SDI regions, so policies to prevent and reduce the burden of the disease should be developed and implemented according to the economic and cultural development of each country. The results of our study can serve as an important reference and provide a scientific basis for the rational allocation of medical resources and health care systems at the regional and national levels.

## AUTHOR CONTRIBUTIONS

**Xiang Qu:** Conceptualization (equal); investigation (equal); methodology (equal); software (equal); visualization (equal); writing – original draft (equal). **Anjie Zheng:** Conceptualization (equal); visualization (equal). **Jie Yang:** Data curation (equal); formal analysis (equal); visualization (equal). **Jinru Zhang:** Investigation (equal); validation (equal). **Hongmei Qiao:** Data curation (equal); resources (equal). **Fan Jiang:** Formal analysis (equal); validation (equal). **Jie Zhao:** Formal analysis (equal); resources (equal). **Chunping Wang:** Data curation (equal); methodology (equal); project administration (equal); supervision (equal); writing – review and editing (equal). **Peng Ning:** Conceptualization (equal); project administration (equal); supervision (equal); writing – review and editing (equal).

## FUNDING INFORMATION

Scientific Research Project of the Chinese Academy of Medical Sciences (IBMS‐2018‐KJHT‐08271810828). The funding body of this research had no role in the design of the study and collection, analysis, and interpretation of data and in writing the manuscript.

## CONFLICT OF INTEREST STATEMENT

The authors declare that they have no conflict of interests.

## Data Availability

The original contributions presented in the study are included in the article. Further inquiries can be directed to the corresponding authors.
